# Long Non-coding RNAs Coordinate Developmental Transitions and Other Key Biological Processes in Grapevine

**DOI:** 10.1038/s41598-019-38989-7

**Published:** 2019-03-05

**Authors:** Garima Bhatia, Shailesh Sharma, Santosh Kumar Upadhyay, Kashmir Singh

**Affiliations:** 1 Department of Biotechnology, Panjab University, 160014 Chandigarh, India; 2 National Institute of Animal Biotechnology (NIAB), D. No. 1-121/1, 4th and 5th Floors, Axis Clinicals Building, Opp. to Talkie Town, Miyapur, Hyderabad, 500 049 Telangana India; 3 Department of Botany, Panjab University, 160014 Chandigarh, India

## Abstract

Long non-coding RNAs (lncRNAs) are transcripts >200 nucleotides that have prominently surfaced as dynamic regulatory molecules. Using computational approaches, we identified and characterized 56,441 lncRNAs in grapevine (*Vitis vinifera*) by harnessing RNA-seq data from 10 developmental stages of leaf, inflorescence, and berry tissues. We conducted differential expression analysis and determined tissue- and developmental stage-specificity of lncRNAs in grapevine, which indicated their spatiotemporal regulation. Functional annotation using co-expression analysis revealed their involvement in regulation of developmental transitions in sync with transcription factors (TFs). Further, pathway enrichment analysis revealed lncRNAs associated with biosynthetic and secondary metabolic pathways. Additionally, we identified 115, 560, and 133 lncRNAs as putative miRNA precursors, targets, and endogenous target mimics, respectively, which provided an insight into the interplay of regulatory RNAs. We also explored lncRNA-mediated regulation of extra-chromosomal genes–i.e., mitochondrial and chloroplast coding sequences and observed their involvement in key biological processes like ‘photosynthesis’ and ‘oxidative phosphorylation’. In brief, these transcripts coordinate important biological functions via interactions with both coding and non-coding RNAs as well as TFs in grapevine. Our study would facilitate future experiments in unraveling regulatory mechanisms of development in this fruit crop of economic importance.

## Introduction

Long non-coding RNAs (lncRNAs) are regulatory non-protein coding transcripts primarily characterized by their length, that is, more than 200 nucleotides in general. These are normally expressed in low levels but in tissue- and cell-type-specific fashion^1^. lncRNAs are predominantly localized in the nucleus^2^ and are known to be poorly conserved at the sequence level during evolution^3^. The biogenesis of lncRNAs is very similar to that of the messenger RNAs (mRNAs). Most lncRNAs are transcribed by RNA polymerase II in eukaryotes and undergo typical post-transcriptional modifications such as 5′ capping, polyadenylation, and splicing, while some are also transcribed by RNA polymerase III^4,5^. Particularly in plants, RNA polymerases IV and V have additionally been observed to transcribe lncRNAs. In fact, an exclusive class of lncRNAs transcribed as products of the plant-specific RNA polymerase V has been reported to be involved in RNA-directed DNA methylation^6,7^. Further, based on their genomic locations with respect to their adjoining protein-coding genes, lncRNAs are categorized into distinct classes such as intergenic, intronic, sense, anti-sense, and bidirectional lncRNAs^3^.

The discovery of lncRNAs in mammals and plants was contemporaneous; however, over the decades, the progress of lncRNA research in the former, particularly in humans, proved trailblazing. An extensive literature supports the involvement of lncRNAs in complex human diseases^8^ such as cancer^9^, cardiovascular disorders like myocardial infarction^10^, and autoimmune disorders like rheumatoid arthritis^11^ and psoriasis^12^. In contrast to the numerous case studies in humans, roles of limited lncRNAs have been revealed in plants. For instance, anti-sense and intronic lncRNAs *COOLAIR* and *COLDAIR*, respectively, epigenetically silence the key flowering repressor FLOWERING LOCUS C (FLC) in the vernalization-dependent pathway^13,14^. Other important lncRNAs are *LDMAR*, *IPS1*, *ENOD40*, *ASCO-lncRNA*, *APOLO*, and *NERDL*, which have been characterized as regulators of biological processes like fertility, phosphate homeostasis, nodule formation, lateral root development, auxin-controlled development, and secondary wood formation, respectively^15–21^. As per recent reviews, genome-wide plant lncRNA research has lately re-emerged owing to the widespread application of the next-generation RNA-sequencing. Presently, thousands of novel lncRNAs have been predicted *in silico* in several plant species in response to different conditions such as stress (biotic and abiotic), nutrient starvation, and hormone treatments^22,23^. Further, well-established studies in animals and few yet promising studies in plants have highlighted the plausible involvement of lncRNAs in tissue development-a vital biological phenomenon^24–30^.

In the present study, we harnessed RNA-seq data for different tissues of *Vitis vinifera* (grapevine) at various developmental stages and identified 56,441 putative lncRNAs via a computational pipeline. *V. vinifera* is an economically and nutritionally important perennial fruit crop, for which considerable genomic and transcriptomic information is available; hence, conducting genome-based global analysis is feasible in the plant. Several “omics” studies in *V. vinifera* have primarily focused on understanding the role and interplay of DE protein-coding genes, metabolites, and proteins at different developmental stages, particularly at key points during mature berry development^31–33^. Through this study, we tried to explore the DE lncRNAs during different developmental stages and identify their possible biological roles in the plant. Additionally, we investigated relationships among the elements of the non-coding genome (like lncRNAs and micro RNAs [miRNAs]) and the protein-coding mRNAs. Since lncRNAs regulate key biological processes in other organisms including various plant species, it has been necessary to investigate their potential functions in different tissues and developmental stages of the most widely cultivated species of grapevine, *V. vinifera*. Our study is a step in this direction and provides information to facilitate the process of understanding the underlying mechanisms of gene expression with emphasis on lncRNAs as new but important players.

## Results

### Genome-wide Identification of lncRNAs in *V. vinifera*

The RNA-seq data (≈200GB) retrieved for leaf, inflorescence, and berry tissues at 10 different developmental stages were assembled into 3,59,570 contigs using Trinity package^34^. Different classical filters were applied to identify the potential lncRNAs. A total of 2,51,439 contigs having open reading frames (ORFs) encoding proteins greater than 100 amino acids in six frame translation were eliminated. The coding potential analyses using CPC^35^ filtered additional 14 transcripts. Homology search filter was applied on the remaining 1,08,117 transcripts by performing BLASTX analysis against the NCBI NR protein database, which ultimately resulted into the identification of final 56,441 transcripts as putative lncRNAs. As a cursory check of our computational approach, we performed standalone BLASTN for these 56,441 putative lncRNAs with respect to lncRNAs available on CANTATAdb^36^ for *V. vinifera* that revealed hits for 7,262 putative lncRNAs, which indicates that our computational approach is reliable. Additionally, this suggests that out of the identified putative lncRNAs, 49,179 lncRNAs are new.

### Characteristics of the Identified *V. vinifera* lncRNAs

Different characteristics of the predicted lncRNAs were examined to understand the trends of their occurrence in the genome. These characteristics were also studied for the mature mRNAs in order to juxtapose the two different transcript categories.

#### Chromosomal Distribution

**Figure 1:**
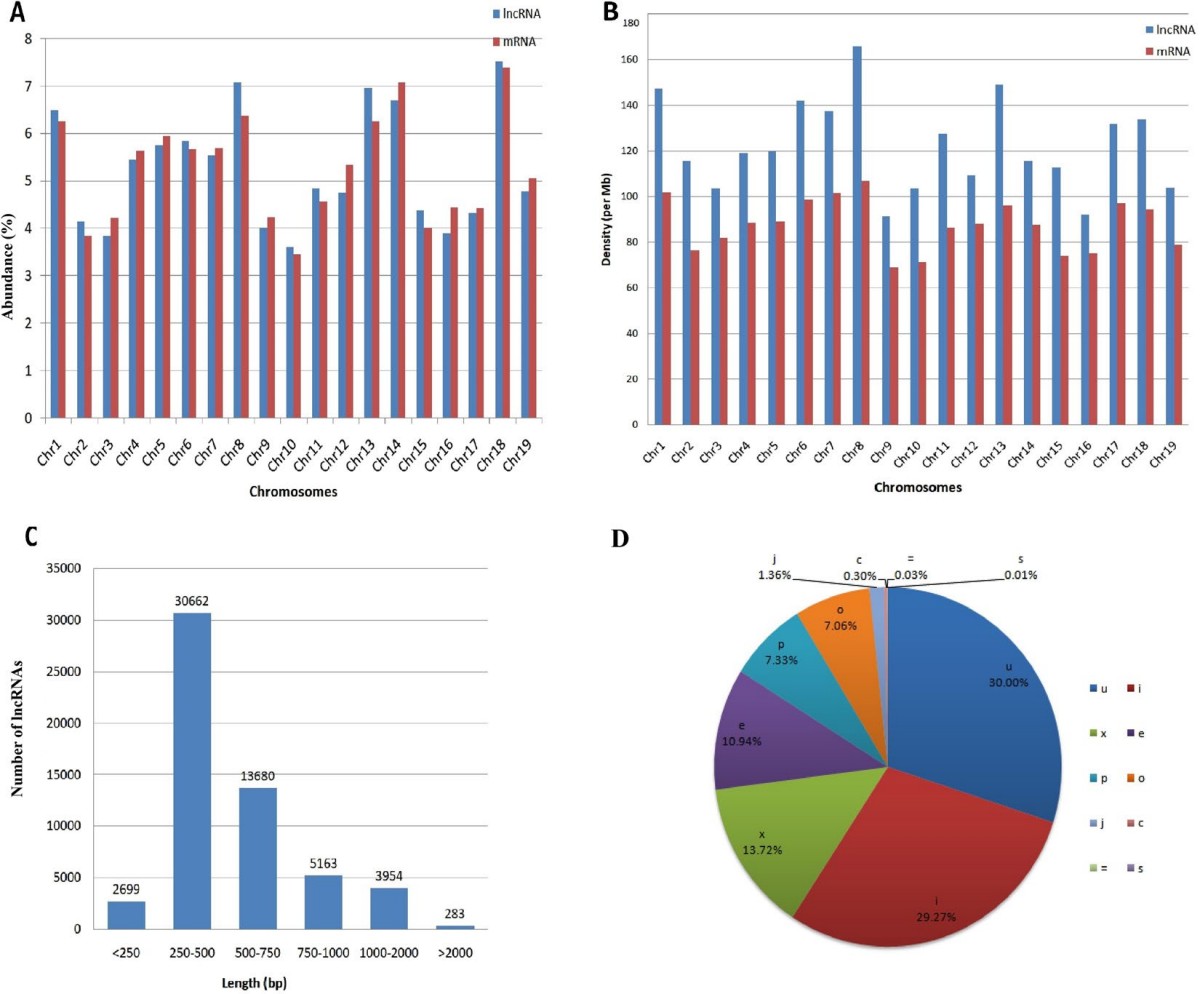
Features of the identified *V. vinifera* lncRNAs (**A**) Chromosome-wise distribution of lncRNAs and mRNAs. The results are depicted as abundance (percentage) of lncRNAs and mRNAs per chromosome (**B**) The density of lncRNAs and mRNAs on different chromosomes. (**C**) Length distribution of 56,441 lncRNAs. (**D**) Classification of lncRNAs based on their genomic locations with respect to that of the protein coding genes. The pie chart depicts the percentage of lncRNAs falling into different categories (that is, class codes as per Cuffcompare-based analysis). The class codes correspond to the following: “=”, complete match with intron chain; “c”, contained; “j”, potentially novel isoform; “e”, single exon transcript overlapping a reference exon that could be a pre-mRNA fragment; “i”, lncRNA falling entirely within a reference intron; “o”, generic exonic overlapping with the reference transcript; “p”, possible polymerase run-on fragment; “u”, unknown intergenic transcript; “x”, exonic overlap with the reference transcript on the opposite strand; “s”, intronic overlap with the reference transcripts possibly due to mapping errors. Chr- chromosome.

Out of 56,441 predicted lncRNAs, we could determine the chromosomal localization of 52,144 lncRNAs, which were unevenly distributed across the 19 chromosomes (Fig. 1A). The highest (7.5%) and lowest (3.6%) proportion of lncRNAs were located on chromosome 18 and chromosome 10, respectively. Similar distribution pattern was observed for the 37,420 mature mRNAs with the highest (7.39%) and lowest (3.45%) proportions on chromosomes 18 and 10, respectively.

#### Chromosomal Density

The chromosomal density analysis revealed that lncRNAs were more densely present on *V. vinifera* chromosomes than the mature mRNAs (Fig. 1B). Chromosome 8 had the highest lncRNA density with 165.61 lncRNAs per Mbp of nucleotides, whereas chromosome 9 had the lowest density with 91.15 lncRNAs per Mbp of nucleotides. Interestingly, the maximum and minimum chromosomal densities for mature mRNAs were also observed on chromosome 8 with 107.02 mRNAs per Mbp of nucleotides and chromosome 9 with 69.11 mRNAs per Mbp of nucleotides, respectively. Moreover, similar density pattern was observed for the both transcript categories despite the differences in the density magnitude.

#### Length Distribution

The length of lncRNAs ranged from 224 bp to 3,910 bp; nevertheless, length of more than half of the lncRNAs (54.3%) varied between 250 to 500 bp (Fig. 1C).

In addition to these characteristics, the lncRNAs were classified based on their genomic locations with respect to those of the neighboring protein-coding genes (Fig. 1D). The majority of the identified lncRNAs, that is, 30%, 29.27%, and 13.72% were classified as intergenic (transcripts mapped to the unknown intergenic regions), intronic (transcripts mapped completely within the introns of the known protein-coding genes), and antisense (transcripts mapped to the exon of a protein-coding gene but on the opposite strand) lncRNAs, respectively.

### Expression Analyses and Estimation of Tissue and Developmental Stage Specificity of the lncRNAs

**Figure 2:**
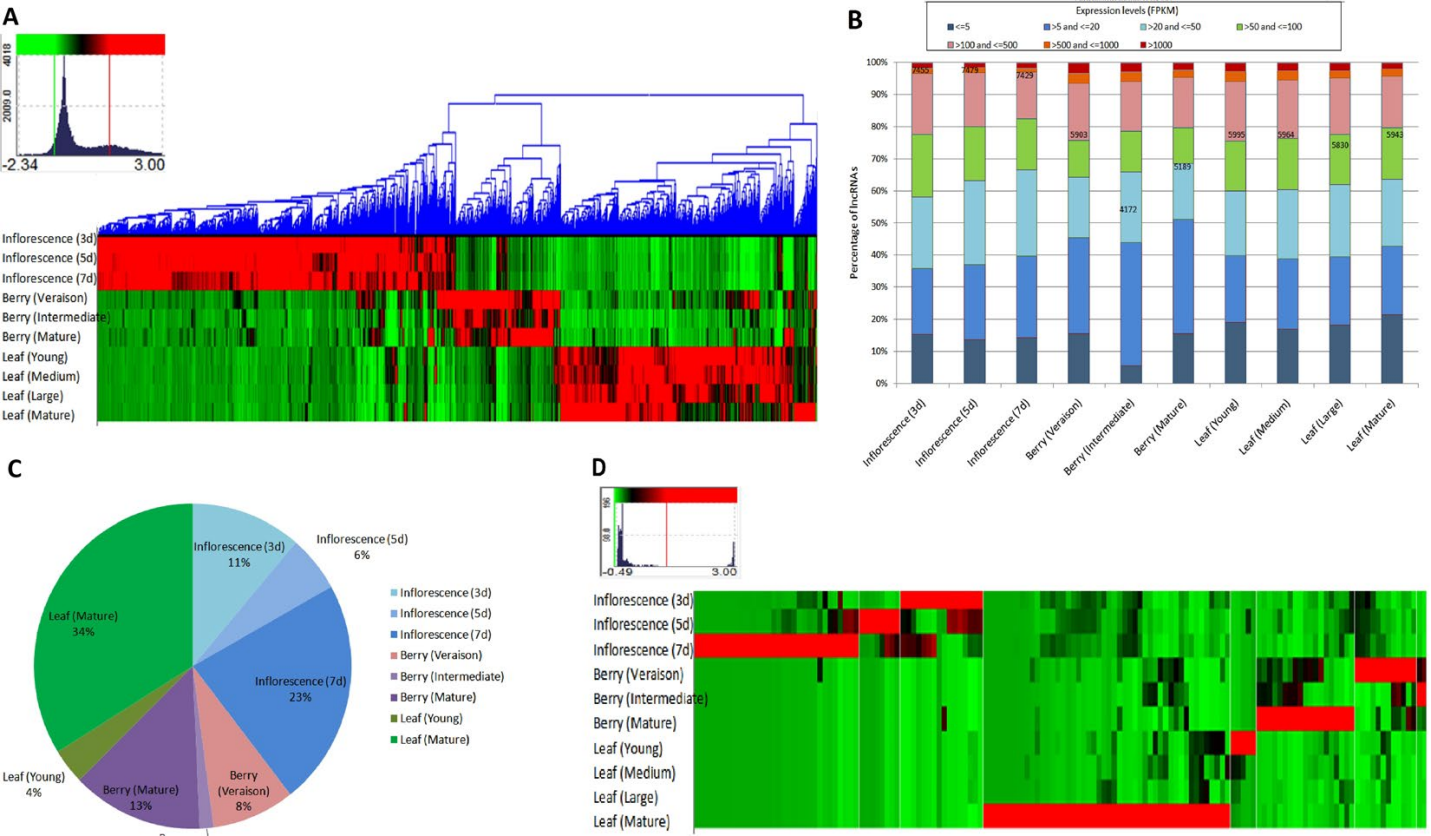
Differentially expressed and tissue- and developmental stage-specific lncRNAs (**A**) Expression profile of 7697 lncRNA transcripts (represented by columns) in 3 tissues and at 10 developmental stages (represented by rows). The color mosaic attached to the dendrogram (x-axis) visualizes the hierarchical clustering result. The arrangement of lncRNAs is according to similar expression levels and hot-spots can be identified in red. The color scale and histogram for the whole data set can be seen in top left corner. (**B**) Distribution of lncRNAs in various categories based on expression levels in different tissues and developmental stages. (**C**) Proportion of lncRNAs exhibiting tissue and developmental stage specificity. (**D**) Expression profiles of 142 tissue- and developmental stage-specific lncRNAs (represented by columns). The color scale and histogram for the whole data set can be seen in top left corner.

To identify the differentially expressed (DE) *V. vinifera* lncRNAs across the inflorescence, berry, and leaf tissues under 10 developmental stages, FPKM values were determined using RSEM^37^ and edgeR^38^ as a part of the Trinity software package. On applying a threshold cut-off of *P*-values (FDR) < = 0.001 and 4-fold change, 7697 DE lncRNAs were identified (Fig. 2A). A maximum of 7479 DE lncRNAs was found in inflorescence tissue at the developmental stage of 5 days post 100% cap-fall. The lowest number of lncRNAs (4172) was expressed in berry tissue at the intermediate stage of ripening, which corresponds to E-L stage 36. On an average, 7454, 5088, and 5933 lncRNAs were expressed in inflorescence, berry, and leaf tissues, which indicated the plausible importance of those lncRNAs in reproductive tissues particularly from flower to fruit development. Further, based on the applied parameters, 5411 DE mature mRNAs were obtained. Of the total mRNAs and lncRNAs studied here, approximately 14.5% and 13.6% transcripts exhibited differential expression patterns across the different tissues and developmental stages, respectively, (Supplementary Fig. 1). Additionally, based on the expression levels, the lncRNAs were categorized into 7 groups: (1) extremely low (FPKM, >0 and <= 5); (2) very low (FPKM, >5 and <= 20); (3) low (FPKM, >20 and <= 50); (4) moderate (FPKM, >50 and <= 100); (5) high (FPKM, >100 and <= 500); (6) very high (FPKM, >500 and <= 1000); and (7) extremely high (FPKM, >1000) (Fig. 2B). More than 70% of the lncRNAs were expressed between moderate and extremely low levels in each tissue/developmental stage. The “high to extremely high expressing” lncRNAs suggested the possibility of a biological rationale in certain tissues at specific stages of development.

Based on the method described by Julien *et al*., we calculated specificity indices for the inflorescence, berry, and leaf tissues at different developmental stages^39^. On applying a cut-off of 0.7 as the threshold specificity index value, a total of 142 highly tissue and developmental stage specific lncRNAs were identified (Fig. 2C). The maximum proportion (34%) of developmental stage-specific lncRNAs was observed in the mature leaf tissue. Moreover, half of the mature leaf-specific lncRNAs belonged to the “high to extremely high expression” groups. Overall, 40% of the tissue-specific lncRNAs were determined in the inflorescence tissue; more than half (57.5%) of them were found to be specific for the stage of 7 days after 100% cap-fall. Likewise, 22% of the tissue-specific lncRNAs were observed in the berry tissue; 59% of them were specific to the mature berry stage. Variable expression levels across the tissues at distinct developmental stages were observed in the expression heatmap of tissue-specific lncRNAs (Fig. 2D).

### Functional Annotation of the lncRNAs

**Figure 3:**
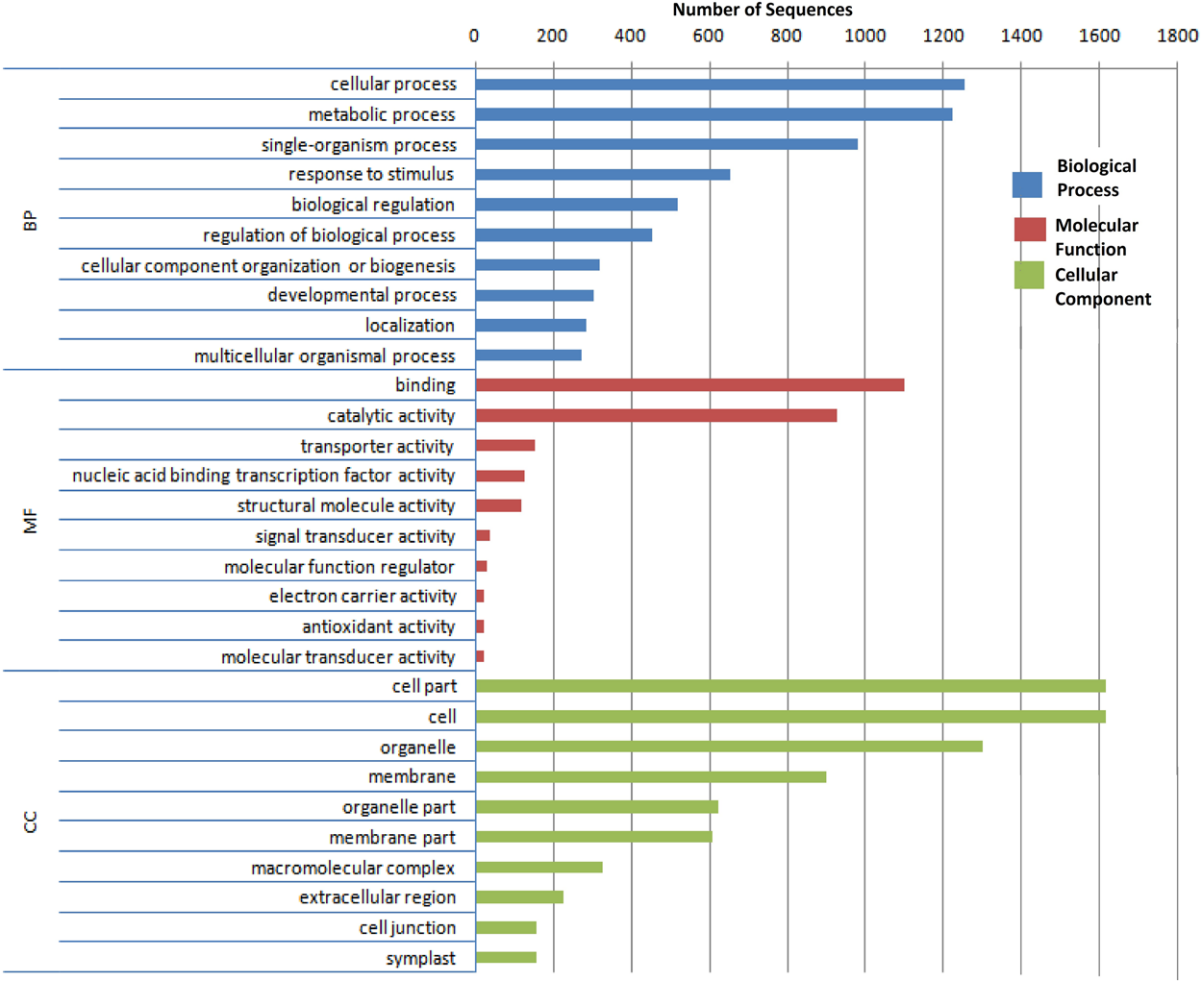
Top gene ontology (GO) Terms showing enrichment for lncRNAs co-expressing with mRNAs: The enrichment is represented in three categories: BP, biological process; MF, molecular function; and CC, cellular component.

In order to annotate the putative functions of the lncRNAs, we conducted co-expression analysis of these transcripts with the mature mRNAs. Firstly, 56,441 lncRNAs and 37,420 mRNAs were filtered to eliminate the transcripts with an average expression <30 FPKM value. Consequently, 9,933 lncRNAs and 6,104 mRNAs were analyzed. Co-expression correlation between lncRNAs and mature mRNAs was calculated using Pearson correlation with R^2^ ≥ 0.9. We found that 6,628 lncRNAs co-expressed with 2,010 mature mRNAs (Supplementary Table 1). GO enrichment analysis was performed for these 2,010 mRNAs using Blast2GO^40^, and nearly 95% of these could be functionally annotated with at least one GO term in the following three categories: cellular component (example, GO:0005634 ‘nucleus’ [30.04% sequences], GO:0005737 ‘cytoplasm’ [70.82% sequences]); molecular functions (example, GO:0005488 ‘binding’ [69.56% sequences], GO:0003824 ‘catalytic activity’ [58.67% sequences]); and biological processes (example, GO:0050896 ‘response to stimulus’ [40.56% sequences], GO:0032502 ‘developmental process’ [18.76% sequences]) (Supplementary Table 2). Figure 3 highlights the top terms for each category. Further data mining on annotation results was conducted using Blast2GO, and enzyme codes (EC) were obtained for the annotated sequences accordingly (Supplementary Fig. 2). The EC distribution pattern revealed that out of the six major EC classes, the maximum co-expressing lncRNA-mRNA pairs belonged to hydrolases, transferases, and oxidoreductases classes. Subsequently, we conducted the pathways enrichment analysis using KEGG pathways database, and the results indicated that lncRNAs are potentially involved in representatives of 121 pathways (Supplementary Table 3). Among these, based on the number of highly enriched enzymes, lncRNAs are likely to be involved in regulation of the following pathways: ‘biosynthesis of antibiotics’, ‘purine metabolism’, ‘starch and sucrose metabolism’, ‘cysteine and methionine metabolism’, ‘glycolysis/gluconeogenesis’, ‘amino sugar and nucleotide sugar metabolism’, ‘porphyrin and chlorophyll metabolism’, ‘phenylalanine, tyrosine and tryptophan biosynthesis’, ‘carbon fixation in photosynthetic organisms’, and ‘flavonoid biosynthesis’. Interestingly, this analysis also revealed the potential involvement of 10 and 14 lncRNAs in regulation of two key processes for the plant: photosynthesis and oxidative phosphorylation, respectively (Supplementary Fig. 3).

### Interactions between lncRNAs and Transcription Factors in *V. vinifera*

**Figure 4:**
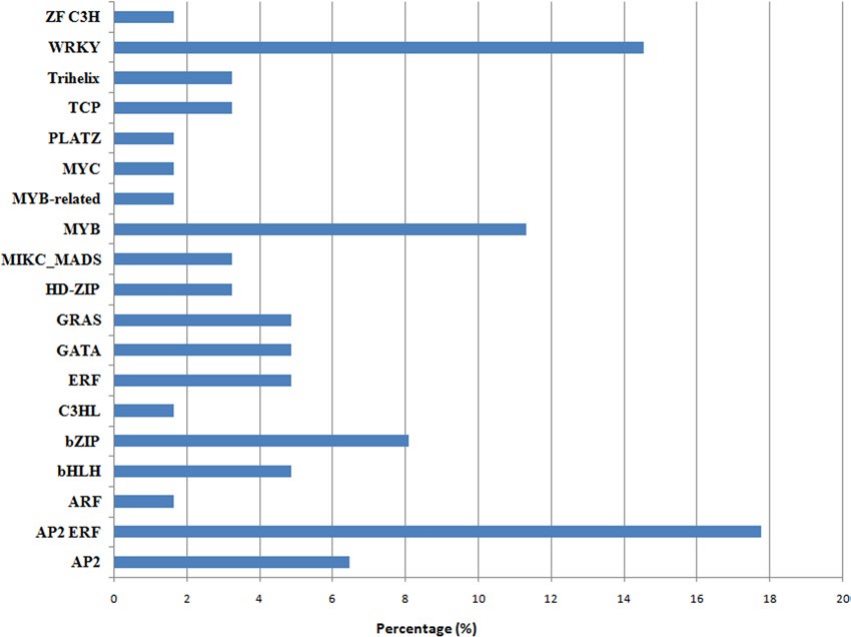
Transcription factor families co-expressing with lncRNAs. The highest percentage of transcription factor families co-expressing with lncRNAs is seen for AP2/ERF superfamily.

Since the sequence information for the TFs known in *V. vinifera* was available at Plant TF database (PlantTFDB) v4.0, we explored the potential association of these regulatory players with the predicted lncRNAs by conducting co-expression analysis. We identified 62 TFs belonging to 19 TF superfamilies and families that were co-expressed with lncRNAs across the different tissues and developmental stages (Fig. 4). Of these, APETALA2/Ethylene Responsive Factor (AP2/ERF) superfamily, which is known to be conservatively widespread in the plant kingdom, was found to be highly enriched (17.7%). The other TFs that were highly co-expressed belonged to WRKY (14.5%) and MYB (11.3%) TF families.

### Interactions of lncRNAs with mRNAs and miRNAs in *V. vinifera*

**Figure 5:**
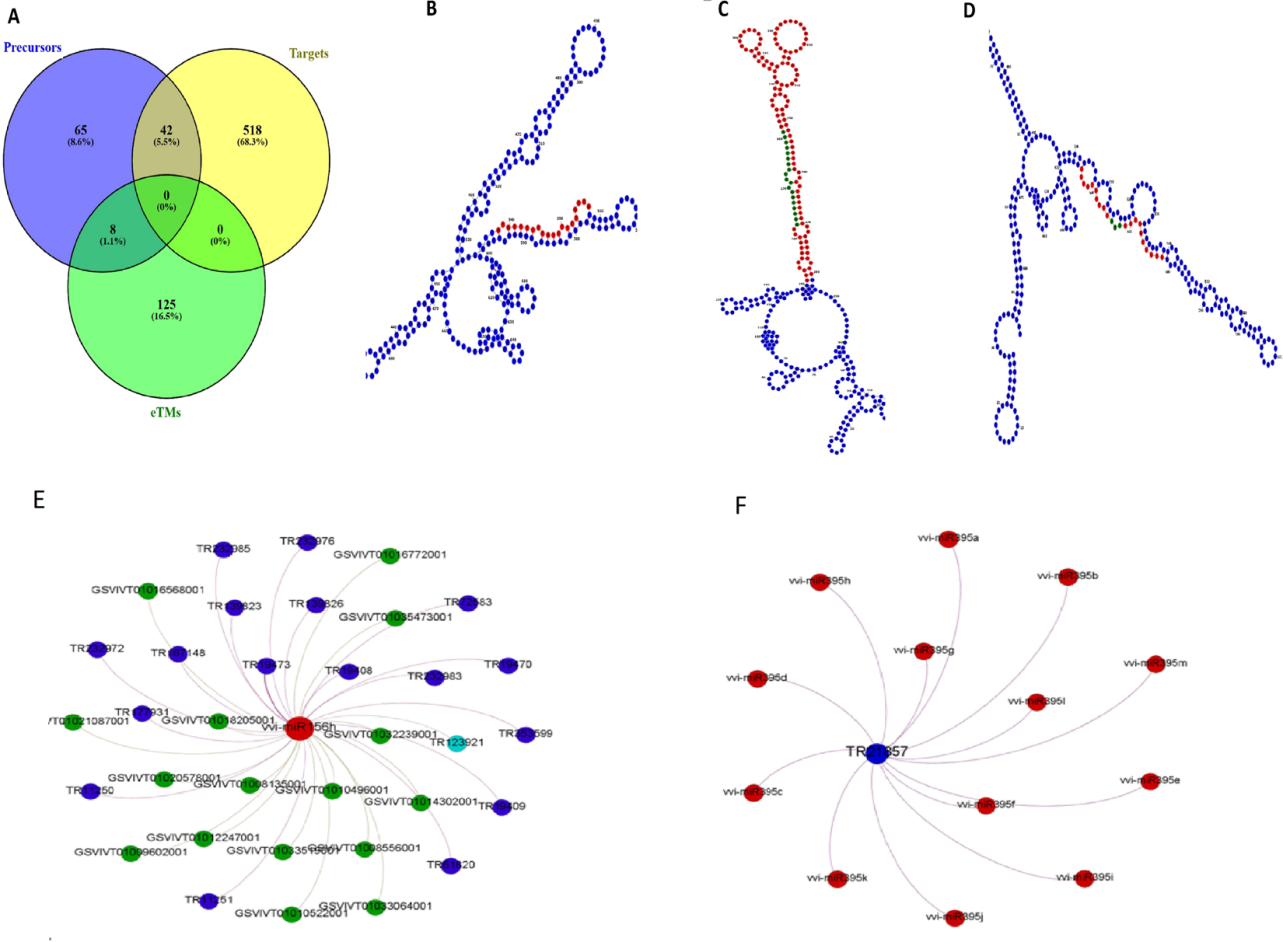
LncRNAs as putative targets, precursors, and endogenous target mimics (eTMs) of miRNAs. (**A**) A Venn diagram showing lncRNAs that can act as: (i) both precursors and targets of miRNAs, and as (ii) both precursors and eTMs of miRNAs. (**B**) *Secondary structure of an lncRNA (TR167148) shown in blue, which acts as a putative target of miRNA (vvi-miR156h) shown in red. (**C**) *Secondary structure of an lncRNA (TR29907) shown in blue, which acts as a putative precursor of miRNA (vvi-miR166a). The precursor (stem-loop) and mature miRNA regions are marked in red and green, respectively. (**D**) *Secondary structure of an lncRNA (TR123921) shown in blue, which acts as a putative eTM for miRNA (vvi-miR156h) shown in red. The characteristic 3-nt bulge is shown in green. *The secondary structures shown in this figure are cropped images used for better display purpose. For viewing the complete image of the secondary structures, refer to Supplementary Fig. 4. (**E**) Interaction network analysis representing a miRNA (red) with multiple lncRNAs (blue) and mRNAs (green). A potential endogenous target mimic (eTM) is marked in cyan. (**F**) Interaction of an lncRNA (blue) with multiple miRNAs (red).

*V. vinifera*-specific mature miRNAs were also downloaded from mirBase^41^ (miRNA database). Using plant small RNA target analysis server, psRNATarget, we identified miRNA target sites in the lncRNAs. Our analyses revealed 560, 115, and 133 lncRNAs as putative micro RNA (miRNA) targets, precursors, and endogenous target mimics (eTMs), respectively (Fig. 5A, Supplementary Table 4). We identified lncRNAs that potentially interact with miRNAs known to play important roles in plant developmental processes such as vvi-miR166, vvi-miR156, vvi-miR172, and vvi-miR319. Figure 5(B–D,E,F) represent the examples of secondary structure prediction and interaction analyses, respectively. The secondary structures shown in Fig. 5 are cropped images used for better display purpose. For viewing the complete image of the secondary structures, refer to Supplementary Fig. 4. The intricacy of the interactome comprising lncRNAs, miRNAs, and mRNAs can be seen in the Supplementary Fig. 5.

### Potential Regulation of Genes of Chloroplast and Mitochondrion by lncRNAs

**Figure 6:**
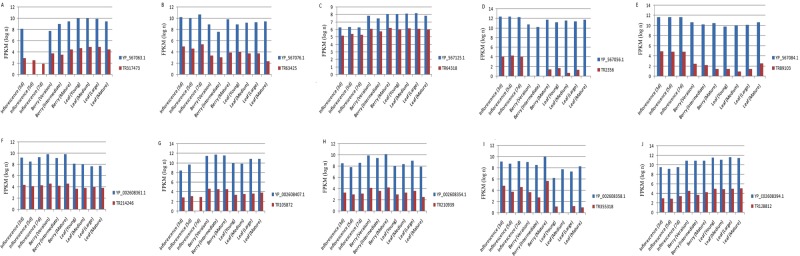
Co-expression patterns of selected lncRNAs and corresponding chloroplast and mitochondrial coding sequences (CDS). (**A**–**E**) depict the co-expression patterns of 5 randomly selected lncRNAs-chloroplast CDS pairs. (**F**–**J**) depict the co-expression patterns of 5 randomly selected lncRNAs-mitochondrial CDS pairs. The names of the lncRNAs and NCBI reference sequence IDs of the CDS are provided in the color legends in each panel. The y-axis corresponds to the natural logarithm of the FPKM values.

In order to inspect whether the lncRNAs shared homology with the extra-chromosomal genome of the plant, we conducted a standalone BLASTN analysis (length of alignment, >200 nt). We obtained 52 and 139 hits for lncRNAs with respect to the chloroplast and mitochondrial genome, respectively. Further, the co-expression analysis of the 56,441 lncRNAs was performed with the 84 chloroplast and 74 mitochondrial CDS, separately. A total of 1,753 and 1590 lncRNAs were found to co-express with 38 chloroplast and 25 mitochondrial CDS, respectively. The highly enriched GO terms associated with the co-expressed chloroplast CDS included photosynthesis (GO:0015979), cellular metabolic process (GO:0044237), and translation (GO:0006412). With respect to the co-expressed mitochondrion CDS, the top GO terms showing very high enrichments included: organonitrogen compound biosynthetic process (GO:1901566), translation (GO:0006412), amide biosynthetic process (GO:0043604), and peptide biosynthetic process (GO:0043043). Intrigued by these findings, we additionally performed the functional annotation of the co-expressing CDS using Blast2GO. The top terms obtained under the biological category for chloroplast and mitochondrion CDS have been shown in Supplementary Fig. 6. Further, ECs were obtained for the annotated sequences (Supplementary Fig. 6). Out of the six major EC classes, the co-expressed chloroplast CDS belonged to four EC classes, oxidoreductases, transferases, hydrolases, and lyases in the decreasing order. Likewise, for the mitochondrion CDS co-expressing with lncRNAs, the EC distribution pattern revealed enrichment for two classes, oxidoreductases and hydrolases. The pathways enrichment analysis indicated that lncRNAs co-expressing with chloroplast and mitochondrion CDS are potentially involved in seven (including ‘purine metabolism’, ‘oxidative phosphorylation’, ‘pyrimidine metabolism’, and ‘thiamine metabolism’) and three pathways (including ‘oxidative phosphorylation’, ‘purine metabolism’, and ‘thiamine metabolism’), respectively (Supplementary Table 3). Figure 6 shows the co-expression patterns of selected lncRNAs and corresponding chloroplast and mitochondrial coding sequences (CDS).

### qRT-PCR Based Expression Analysis of lncRNAs

**Figure 7:**
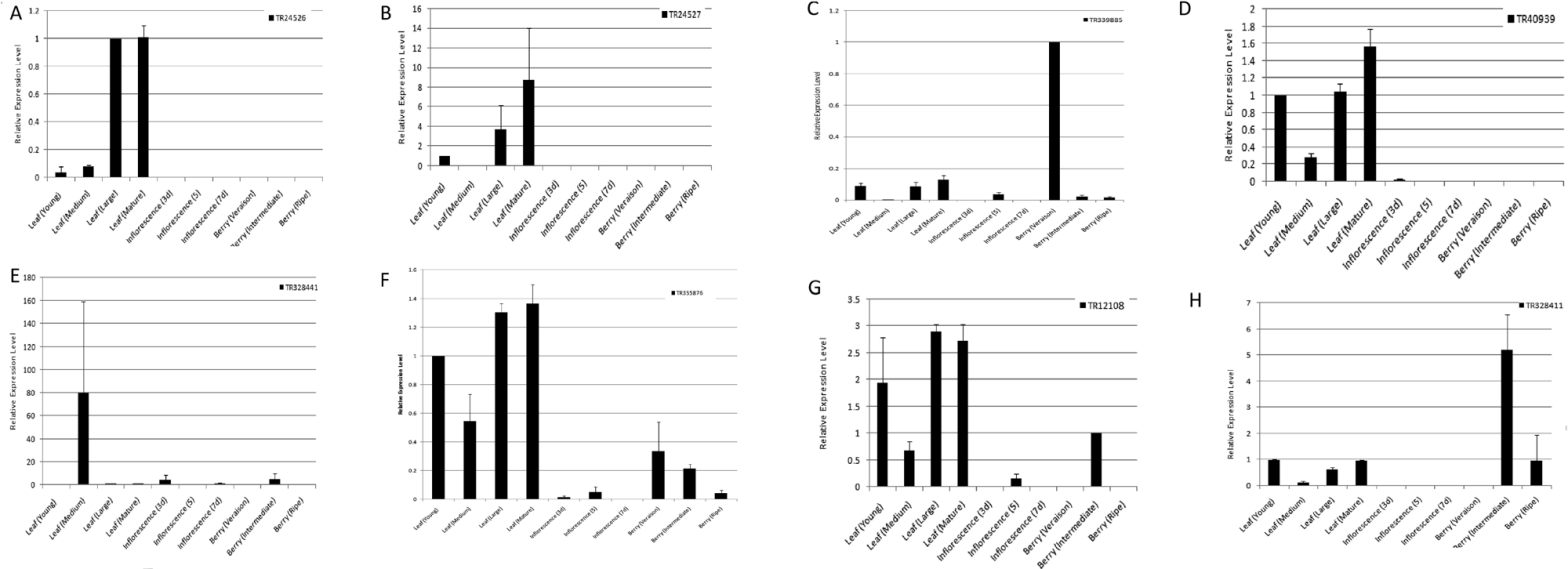
Relative expression analysis of selected lncRNAs using quantitative real time polymerase reaction. Elongation Factor-1 Alpha (*EF1α*) and actin7 (*ACT7*) (NCBI reference sequence ID: XM_002284888.3 and XM_002282480.4) were used for normalization of gene expression. Error bars represent the standard errors of the triplicates.

Of the highly expressing (FPKM > 100) tissue/developmental stage-specific lncRNAs, eight were randomly selected to validate their expression profile across different developmental stages of leaf, inflorescence, and berry tissues using quantitative real time polymerase chain reaction (qRT-PCR). We observed similar trends of expression across tissues as those seen using the RNA-seq data (Fig. 7). For instance, lncRNA TR339885 was more expressed in berry compared to the other two tissues during both qRT-PCR and RNA-seq data based analysis (Fig. 7C). Likewise for lncRNA TR40939, it was observed to be more expressed in the leaf tissue at the mature stage based on both the analyses (Fig. 7D).

## Discussion

Diverse studies have been conducted at the genomic, transcriptomic, proteomic, and metabolomic levels to understand growth, development, and the complicated process of berry ripening in *V. vinifera*^42–45^. Consequently, huge volumes of data have also been generated by the grapevine research community, which need to be carefully mined to unleash the potential players of gene regulation^46^. Like the popularly studied short non-coding RNAs (such as miRNAs, siRNAs, and piRNAs), lncRNAs have emerged as important regulators of gene expression in mammals and have been extensively studied at the tissue and cell levels under normal and disease conditions^8–12^. However, lncRNA research in plants has only recently gained momentum. In this study, we used computational approach to identify lncRNAs in *V. vinifera* and investigated their potential role in regulation of plant development and other processes. The identified lncRNAs were found to express specifically/differentially across the tissues and developmental stages, and their expression patterns were in sync with that of certain mature mRNAs and TFs functionally annotated to be involved in developmental and other biological processes. Moreover, the interaction analyses of *V. vinifera* miRNAs with the predicted lncRNAs revealed the potential regulation of the targets of the former by the latter.

While compiling the findings of the current study, we came across a recent study by Harris *et al*. in which the authors had demonstrated that the transcriptome assembly of single *V. vinifera* cultivar is insufficient to completely annotate *V. vinifera* reference genome (based on Pinot Noir-derived PN40024)^47^. Interestingly, the authors re-annotated the reference genome based on *de novo* transcriptome assembly of another cultivar, Riesling (recovering about 60% of the Riesling transcriptome) and also identified 3210 putative lncRNAs in the cultivar. With their aim to define varietal differences in the plant at the transcriptome level, the authors have focused on cultivar-specific protein-coding RNAs and lncRNAs. However, in the present study, we harnessed grapevine transcriptomic data from three tissues and ten developmental stages to identify 56,441 putative lncRNAs and understand the importance of their existence in this fruit crop. With the primary focus on exploring as many as possible *V. vinifera* lncRNAs, we as well adopted the *de novo* approach of assembling the transcriptome; however, the assembly was based on RNA-seq data from at least three different cultivars, Summer Black, Thompson seedless, and Muscat blanc (Supplemenatry Table 4). The lncRNAs were analyzed to gain an overall picture of the several different ways in which these transcripts could regulate important biological processes in *V. vinifera* such as development.

Foremost, the identified lncRNAs were characterized *in silico*, and it was observed that these were distributed unevenly but densely across the 19 chromosomes. Similar trends of chromosomal distribution were observed for cucumber and tomato lncRNAs^48,49^ suggesting that these can be transcribed from anywhere in the genome. Further, based on digital expression estimation, it was observed that though their abundance varied from low to moderate levels, grapevine lncRNAs were significantly differentially expressed (*P-*value < = 0.001 and 4-fold change) across inflorescence, berry, and leaf tissues at distinct developmental stages. A maximum number of DE lncRNAs was identified in the reproductive tissues; most of them were inflorescence-specific. Interestingly, the maximum proportion of developmental stage-specific DE lncRNAs was determined in the mature leaf stage. Kim and Sung^50^ have previously highlighted the potential of spatially and temporarily differentially expressed lncRNAs in regulating important developmental programs in eukaryotes. Based on digital expression analysis in plants such as *Gossypium arboretum* (cotton), *Cicer arietinum* (chickpea), *Fragaria vesca* (woodland strawberry), and *Morus notabilis* (mulberry), tissue-specific lncRNAs have been reported to be involved in fiber development, flower development, fruit and flower development, and floral organ and root development, respectively^27–29,51^. The present study is the first attempt to identify tissue- and developmental stage-specific lncRNAs in grapevine.

We conducted co-expression analysis to assign a tentative function to the grapevine lncRNAs with respect to the mature mRNAs, and functions of more than 6,000 co-expressing lncRNAs could be deduced using this strategy. The analysis revealed that lncRNAs co-expressed with mature mRNAs that code for enzymes involved in different biosynthetic, primary, and secondary metabolic pathways; for instance, enzymes involved in berry ripening. Grapes are non-climacteric fruits and the initiation of ripening is not controlled by a master switch (like in the case of climacteric fruits where ethylene plays a central and definite role). It has been suggested that abscisic acid (ABA) promotes ripening along with interplay of other hormones^52,53^. Particularly, ABA levels tend to increase at veraison (EL-35) as genes involved in ABA biosynthesis are more expressed^54^. Genes encoding 9-*cis*-epoxy-carotenoid dioxygenase (NCED) and zeaxanthin epoxidase (ZEP), which are two crucial plastidial enzymes in ABA biosynthesis, have been reported to be up-regulated at the EL35 berry stage in grapevine^45,55^. Further, it has been observed that during the later stages of ripening (EL-36 onwards), ABA synthesis is not induced; however, ABA-regulated processes (such as signaling networks) are activated^53,54^. In sync with these studies, we observed the relative expression patterns of grapevine lncRNAs co-expressing with NCED and ZEP (Supplementary Fig. 7). Co-expressing lncRNAs were found to be up-regulated at the EL-35 stage of berry development. Further investigation of these lncRNAs can pave ways into deeper understanding of the mechanisms of berry ripening and can help to resolve the complications of transcriptional reprogramming in other non-climacteric fruits, as well.

Likewise, based on pathway enrichment analysis, grapevine lncRNAs were found to be associated with carotenoid biosynthesis. Interestingly, carotenoids (a subgroup of secondary metabolites- isoprenoids) can act as precursors for ABA, which further regulates plant development. Besides, it has been observed in an independent study that transcriptional regulation of carotenoid biosynthesis pathway genes controls carotenoid content during different developmental stages of fruit ripening^56^. Furthermore, grapevine lncRNAs were found to be associated with phenylpropanoid, flavonoid, and stilbenoid biosynthesis, indicating their potential as regulators of secondary metabolic pathways in the plant (Supplementary Fig. 8). Recently, efforts have been made in this direction by constructing integrated networks^57^. Wong and Matus identified new TFs, miRNAs, and one lncRNA that regulated the expression of stilbene synthase (*SBS*) genes. The present study highlights the potential of lncRNAs in regulation of several secondary metabolites in the plant (Supplementary Table 3). In future, experiments can also be directed towards understanding the underlying regulatory mechanisms of these metabolites.

TFs are instrumental players in regulatory networks. As modular proteins, they interact with both coding and non-coding genes, and are capable of controlling developmental transitions^58^. Such transitions require chromatin re-organization, and TFs in union with chromatin regulators modulate the gene expression during plant development. However, these interactions leading to repression and activation of target genes are not completely understood. Functional analyses of human lncRNAs (like *XIST*) have revealed that these transcripts can bind and mediate recruitment of chromatin-modifying complexes, thereby, acting in *cis* to regulate the target gene expression^59–61^. Likewise, in *Arabidopsis*, lncRNAs (like *COLDAIR*) have been identified that mediate epigenetic silencing of the target genes by directing chromatin modifications and regulate important developmental processes, such as flowering^13,62^. Such lncRNAs that act as scaffolds for chromatin-modifying complexes are not only involved in gene repression but have also been reported as enhancers of expression^63,64^. Thus, lncRNAs are potential links that can help us better understand the molecular dynamics of epigenetic and transcriptional reprogramming during plant development. We observed important families of TFs that co-expressed with grapevine lncRNAs across different developmental stages. Particularly, AP2/ERF superfamily was found to be highly enriched. The members of this family have been reported to be specifically differentially expressed in a study based on transcriptomic analysis of grape berry at different developmental stages^31^. Moreover, other highly co-expressed TFs observed by us, such as WRKY, MYB, bHLH, and Trihelix, were also reported to express differentially in young, veraison, late-veraison, and ripe berries. Different groups of *VvWRKY* family of genes have been reported to be expressed specifically in leaf tissues during senescence^65^, while some have been associated with pathogen defense in ripe berries as the tissue at that stage becomes more prone to fungal attacks^31^. These findings indicate the involvement of grapevine lncRNAs in common developmental and/or metabolic pathways as those associated with TFs in the plant.

In our study, not all the identified lncRNAs were found to co-express with the mRNAs as the former have been reported to function through diverse mechanisms. Moreover, developmental switches are controlled by several key regulators, for instance, TFs, miRNAs, chromatin regulators, etc.^58^; hence, further strategies were developed to mine as many as possible potential roles of grapevine lncRNAs, especially in regulating plant development. Therefore, we studied the interaction of lncRNAs with miRNAs and observed that lncRNAs are potential targets, precursors, and eTMs of the latter. Target mimicry, as an lncRNA-mediated miRNA regulatory mechanism, has been reported in plants like *Arabidopsis*^16^ and *Populus*^66^. We identified several putative eTMs, which can regulate TFs via sequestration/sponging of miRNAs. For instance, lncRNA ‘TR123921’ acts as an eTM for miRNA ‘vvi-miR156h’. The original target for this miRNA has been predicted as Squamosa Promoter Binding Protein (SBP), which are TFs known to be involved in inflorescence development and have also been recently reported in Chinese wild *Vitis* as regulators of floral transition. High expression levels of this eTM were observed in different stages of inflorescence as compared to the leaf and berry tissues. This reflects the lncRNA-mediated positive regulation of the target genes of miRNAs and their impact on plant development. The miRNA family 156 has been known to be associated with SPL TFs in other plants like *A. thaliana*, *O. sativa*, *S. lycopersicum*, and *Z. mays* and affect floral development and plant transition from juvenile to adult stage^67–71^.

Grapevine mitochondrial genome is one of the largest sequenced plant organelle genomes^72^, but the role of lncRNAs in regulation of the mitochondrial genes is unknown. We explored the potential role of lncRNAs in regulation of extra-chromosomal genes, that is, mitochondrial and chloroplast CDS and identified co-expressing lncRNAs that were associated with key biological processes such as photosynthesis, oxidative phosphorylation, and purine/pyrimidine metabolic pathways. Our findings support the feasibility of an additional layer in the regulation of extra-nuclear genes in plants. Further studies aiming at understanding the biogenesis and mechanisms of regulation of these lncRNAs can help solve the mysteries of plant mitogenome and plastome. Studies in mammals have shown that though nucleus is the site of biogenesis of several lncRNAs, these transcripts can dwell in the mitochondrion to facilitate the coordinated signaling system and regulate mitochondrial functions^73,74^.

In this study, we have observed the numerous ways through which lncRNAs are potentially involved in regulation of developmental transitions and other key processes in *V. vinifera*. Besides the *in silico* analyses, qRT-PCR results have highlighted the differential expression patterns of selected lncRNAs across different tissues and developmental stages. These experimentally validated lncRNAs will now further be studied to understand the molecular mechanisms of their action.

## Conclusion

We used a computational pipeline to identify 56,441 putative lncRNAs in *V. vinifera* from RNA-seq data. Further, we identified DE lncRNAs across inflorescence, berry, and leaf tissues at different developmental stages; of which, some transcripts exhibited tissue- and developmental stage-specificity. We observed that 22% of the tissue-specific lncRNAs were highly expressed in the berry, particularly at the mature fruit and veraison stages. Moreover, mature leaf-specific lncRNAs were highly expressed, which draws our attention to explore the possible roles lncRNAs could play in vegetative tissues as well. Co-expression analysis-mediated functional annotation primarily revealed the association of grapevine lncRNAs with development, biosynthetic pathways, and secondary metabolic pathways. Interaction of lncRNAs with miRNAs as their putative precursors, targets, and endogenous target mimics enabled us to propose the plausible mechanism of action for the former. TFs have been known to regulate developmental transitions in grapevine; however, their underlying mechanisms of action are not fully understood. Co-expressing TFs and lncRNAs can be further studied to understand their interaction with chromatin modifiers in repressing and activating genes during different developmental stages of the plant. Lastly, our findings draw attention towards the potential of lncRNAs to regulate extra-nuclear genes in plants. Overall, our results highlight the importance of lncRNAs in coordinating developmental transitions and other biological processes in grapevine.

## Methods

### Transcriptomic Data Collection

RNA-seq data pertaining to different tissues such as leaf (young, medium- and large-sized, and mature), inflorescence (3, 5, and 7 days after 100% cap-fall), and berry (veraison, intermediate, and mature) of *V. vinifera* were obtained based on published studies^75–77^ using National Center for Biotechnology Information (NCBI) Sequence Read Archive (SRA) (http://www.ncbi.nlm.nih.gov/sra). The details of the RNA-seq data collected from NCBI-SRA database have been included in Supplementary Table 5.

### A Computational Approach for Identification of lncRNAs Using the Collected RNA-seq Data

**Figure 8:**
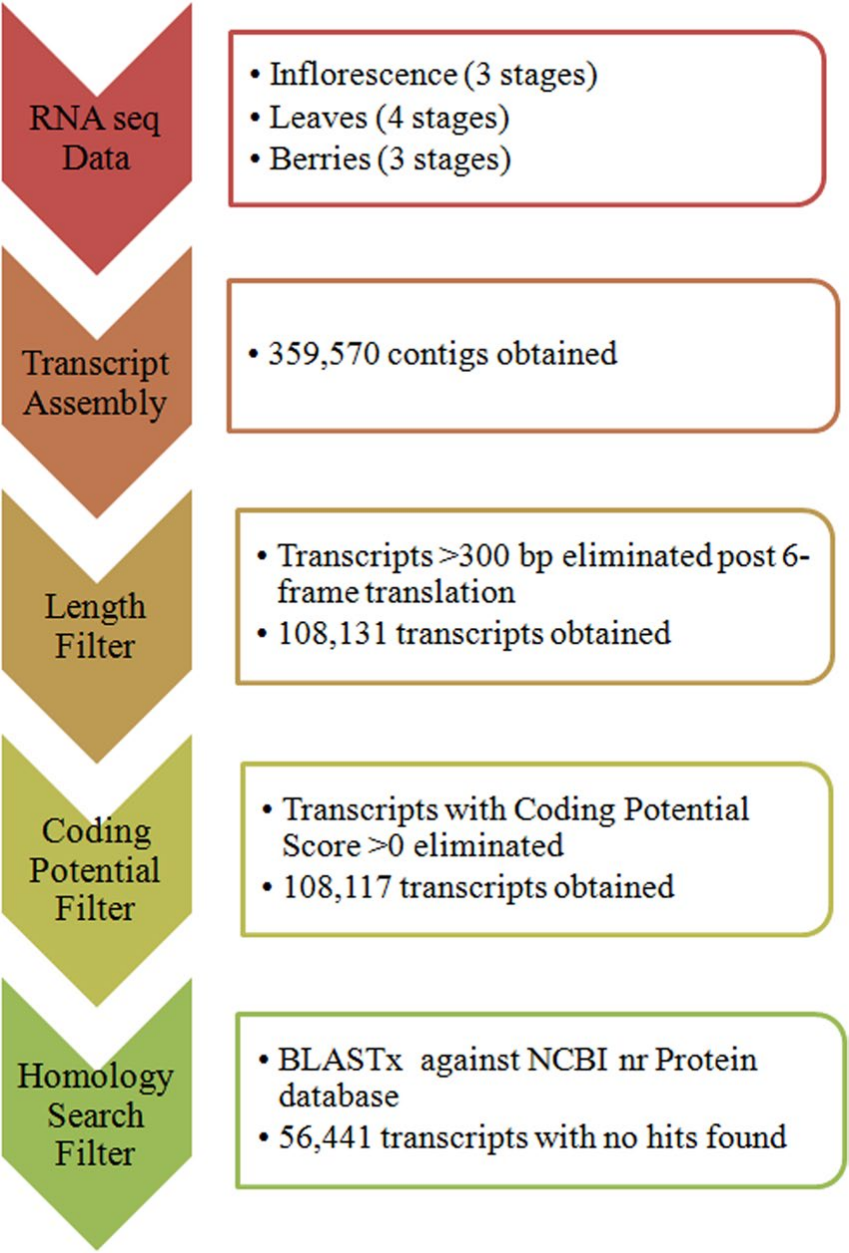
Systematic representation of the computational approach for identification of lncRNAs in grapevine.

A computational pipeline was developed to identify putative lncRNAs using Trinity package^34^ as shown in Fig. 8. After the final assembly, which included removal of low-quality reads and adapter-containing sequences, a six-frame translation was conducted for the resulting contigs, and those coding for proteins greater than 100 amino acids in length were excluded. Next, the coding potential of these putative non-coding transcripts was evaluated using Coding Potential Calculator (CPC)^35^, and only the transcripts with scores less than 0 were used for subsequent analysis. The remaining transcripts were searched against the NCBI non-redundant (NR) protein database by BLASTX to exclude transcripts with significant homology to the known proteins. The transcripts for which no hits were obtained were considered. In order to verify our prediction approach, we compared our putative lncRNAs to the already reported lncRNAs from CANTATAdb^36^ by performing standalone BLASTN.

### *In silico* Characterization and Classification of the Putative lncRNAs

The chromosome sequences of *V. vinifera* were downloaded from NCBI Nucleotide database, and standalone BLASTN analysis was conducted for lncRNAs with the following parameters: per cent identity >90 and e-value < 1e-10. The results were compared to the data available for 37,420 coding sequences (CDS), that is, the mature mRNA transcripts of the plant. These CDS were also downloaded using NCBI resources. For classification of the identified lncRNAs based on their genomic locations relative to that of the neighboring protein-coding genes, Cuffcompare program bundled with Cufflinks package v2.2.1^78^ was used. The classification was aided by the “class codes” generated in the output with respect to the reference protein-coding genes (**see** Supplementary Data 1). The GTF-formatted files used as input for this analysis are provided in the Supplementary Data 2 and 3.

### Differential Expression Analysis

The collected RNA-seq data (as discussed above) from 3 tissues and 10 developmental stages were used to determine the expression levels of both the putative lncRNAs and mature mRNA transcripts. These were quantified as number of RNA-seq fragments per kilobase of transcript effective length per million fragments mapped to all transcripts (FPKM) using RNA-Seq by Expectation-Maximization (RSEM) software that was bundled with Trinity package v2.4.0. To study the DE transcripts across the samples, the Empirical analysis of Digital Gene Expression (edgeR) was invoked, and those DE transcripts that exhibited at least 4-fold change at *P*-values (false discovery rate [FDR]) < = 0.001 in any of the pair-wise sample comparisons were selected. The expression profiles of lncRNAs and mature mRNAs were compared across the different samples with the aid of heat maps developed using Hierarchical Clustering Explorer v3.5^79^ (http://www.cs.umd.edu/hcil/hce).

### Tissue and Developmental Stage Specificity Estimation

To identify the lncRNAs that were specifically expressed in a given tissue and developmental stage, we calculated an index of tissue/developmental stage specificity for each transcript based on respective expression values estimated as FPKM. These indices were calculated based on the method described by Julien *et al*. and recently also applied by Shumayla *et al*.^30,39^. Briefly, the specificity index has been defined as the quotient of consensus expression value of a transcript and the sum of consensus expression values in all tissues and developmental stages. The resulting index ranges from 0 to 1 and corresponds to housekeeping and tissue-/developmental stage-specific lncRNAs accordingly. We used 0.7 as the threshold value of the index to estimate tissue and developmental stage specificity of the lncRNAs.

### Functional Annotation of the Predicted lncRNAs

Prediction of lncRNA functions was carried out based on co-expression analysis of mature mRNAs and lncRNAs as reported previously in some studies^30,80^, that is, identification of the coding and non-coding transcripts co-expressing in different tissues and developmental stages. The expression data (in the form of FPKM) for both mature mRNAs and lncRNAs that were obtained as described above were fed into CoExpress v.1.5 tool, filtered by removing transcripts with the average expression <30, and analyzed following the instructions provided in the corresponding user manual^81^. The following parameters were applied: measure, Pearson correlation; correlation power, 1; filtering threshold, 0.9; and number of runs for bootstrapping, 100. Next, Blast2Go was used to perform gene ontology (GO) enrichment for the mRNAs co-expressing with lncRNAs^40^. Further, pathway enrichment analysis was conducted for the mRNAs co-expressing with lncRNAs to gain insight into the potential pathways being regulated by using Kyoto Encyclopedia of Genes and Genomes (KEGG)^82^ Pathways database.

### Interaction of lncRNAs with Transcription Factors (TFs)

The CDS of the known TFs for *V. vinifera* available in the Plant TF database v4.0^83^ (PlantTFDB) were downloaded, and co-expression analysis (as described above) was conducted to identify the co-expressing TF-lncRNA pairs with similar parameters.

### Interaction of lncRNAs with Other RNAs

For understanding the interaction between the short ncRNAs (miRNAs) and lncRNAs, *V. vinifera*-specific 186 mature miRNAs were downloaded from miRNA database (miRBase). To predict the miRNA target sites in lncRNAs, plant small RNA target analysis server^84^ (psRNATarget) was used with default parameters.

Furthermore, in order to explore the role of lncRNAs as precursors of *V. vinifera* miRNAs, 163 stem-loop sequences of the latter were downloaded from miRBase. Standalone BLASTN was performed to compare the downloaded sequences with the lncRNAs. In order to identify the lncRNAs acting as eTMs, TAPIR^85^ (http://bioinformatics.psb.ugent.be/webtools/tapir/) was used with mfe_ratio >= 0.7.

The secondary structures based on minimum free energy were analyzed using Vienna RNAfold web server (http://rna.tbi.univie.ac.at/)^86^.

To understand the relationships among the coding and non-coding RNAs, interaction network analysis was conducted using Gephi (https://gephi.org/)^87^.

### Potential Regulation of Extra-chromosomal Genetic Material by lncRNAs

The extra-chromosomal genome of *V. vinifera*, that is, nucleotide sequences of the chloroplast and mitochondrion were retrieved using the NCBI Nucleotide database, and standalone BLASTN analysis was conducted with respect to the lncRNAs. Further, using NCBI resources, 84 and 74 chloroplast and mitochondrial CDS were downloaded, and the DE analysis (as described above) was conducted to study their expression patterns across the different tissues and developmental stages. Next, co-expression analysis was conducted in the aforementioned manner to identify the co-expressing chloroplast- and mitochondrial-lncRNA pairs by using similar parameters.

### qRT-PCR Based Expression Analysis of lncRNAs

To validate the expression of lncRNAs across different tissues, leaf (young- 2 weeks, medium- 5 weeks, large- 7 weeks, mature 10 weeks), inflorescence (3, 5, and 7 days after 100% cap-fall), and berry (veraison, intermediate, and mature) samples of *V. vinifera* cv. Thompson seedless were collected during the ongoing season in March-June 2018. Total RNA was isolated using the protocol by Ghawana *et al*.^88^, and the quantity and integrity of the RNA samples were analyzed by measuring 260/280 nm ratios using Nanodrop spectrophotometer and by 1.2% agarose gel electrophoresis, respectively. cDNA was prepared using Bio-Rad iScript^TM^ Select cDNA synthesis kit. The primers for qRT-PCR were designed using Primer3 Input software (Supplementary Table 6). PCR amplifications were carried out using Bio-Rad CFX96™ Real-Time PCR system. Elongation Factor-1 Alpha (*EF1α*) and actin7 (*ACT7*) (NCBI reference sequence ID: XM_002284888.3 and XM_002282480.4, respectively) were used as internal control genes for normalization of gene expression. 2^−ΔΔCT^ method was used to estimate the relative gene expression^89^. All the experiments were conducted in triplicates.

## Supplementary information

figure

LaTeX Supplementary File

LaTeX Supplementary File

LaTeX Supplementary File

LaTeX Supplementary File

LaTeX Supplementary File

LaTeX Supplementary File

LaTeX Supplementary File

LaTeX Supplementary File

LaTeX Supplementary File
