# Supplementary material for: Long Non-coding RNAs Coordinate Developmental Transitions and Other Key Biological Processes in Grapevine
Source: Sci Rep. 2019 Mar 5;9:3552. doi: 10.1038/s41598-019-38989-7 (PMC6401051; doi:10.1038/s41598-019-38989-7)
Supplement: Supplementary file 1 — figure [file 41598_2019_38989_MOESM1_ESM.pdf]

Long Non-coding RNAs Coordinate Developmental Transitions and Other Key Biological Processes in Grapevine

Authors: Garima Bhatia[1], Shailesh Sharma[2], Santosh Kumar Upadhyay[3], Kashmir Singh[1*]

[1]Department of Biotechnology, Panjab University, Chandigarh, India-160014.

[2]National Institute of Animal Biotechnology (NIAB), D. No. 1-121/1, 4th and 5th Floors, Axis Clinicals Building, Opp. to Talkie Town, Miyapur, Hyderabad, Telangana, India - 500 049.

[3]Department of Botany, Panjab University, Chandigarh, India-160014.

*Corresponding Author: Dr. Kashmir Singh, Associate Professor, Department of Biotechnology, BMS Block I, Panjab University, Sector 25, Chandigarh, India-160014. Email: kashmirbio@pu.ac.in; Tel: +91-172-2534085

This PDF Document Contains Supplementary Figures 1 to 8

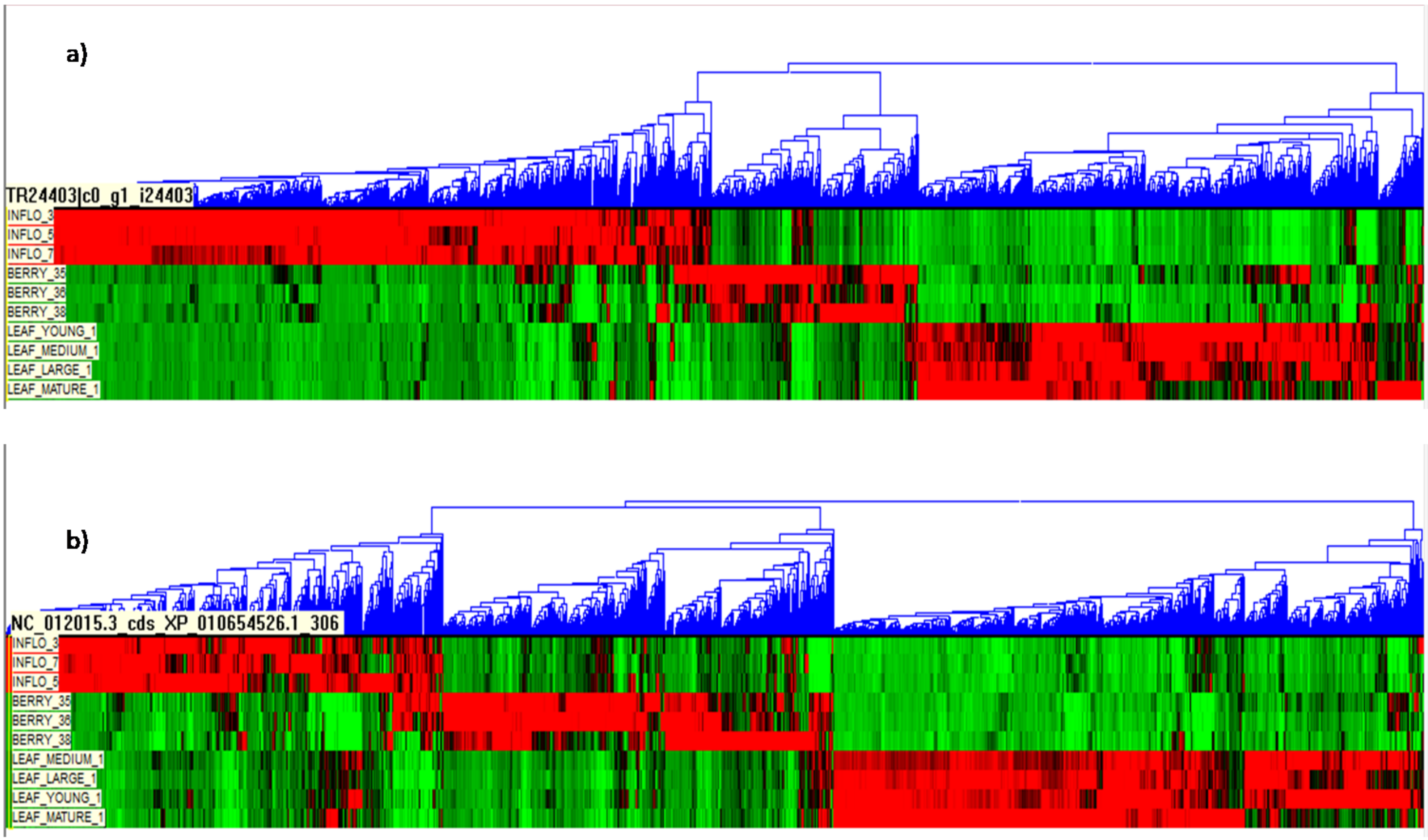

Supplementary Figure 1: Comparison of expression profiles of A) lncRNAs and B) mature mRNA transcripts in 3 tissues and at 10 developmental stages.

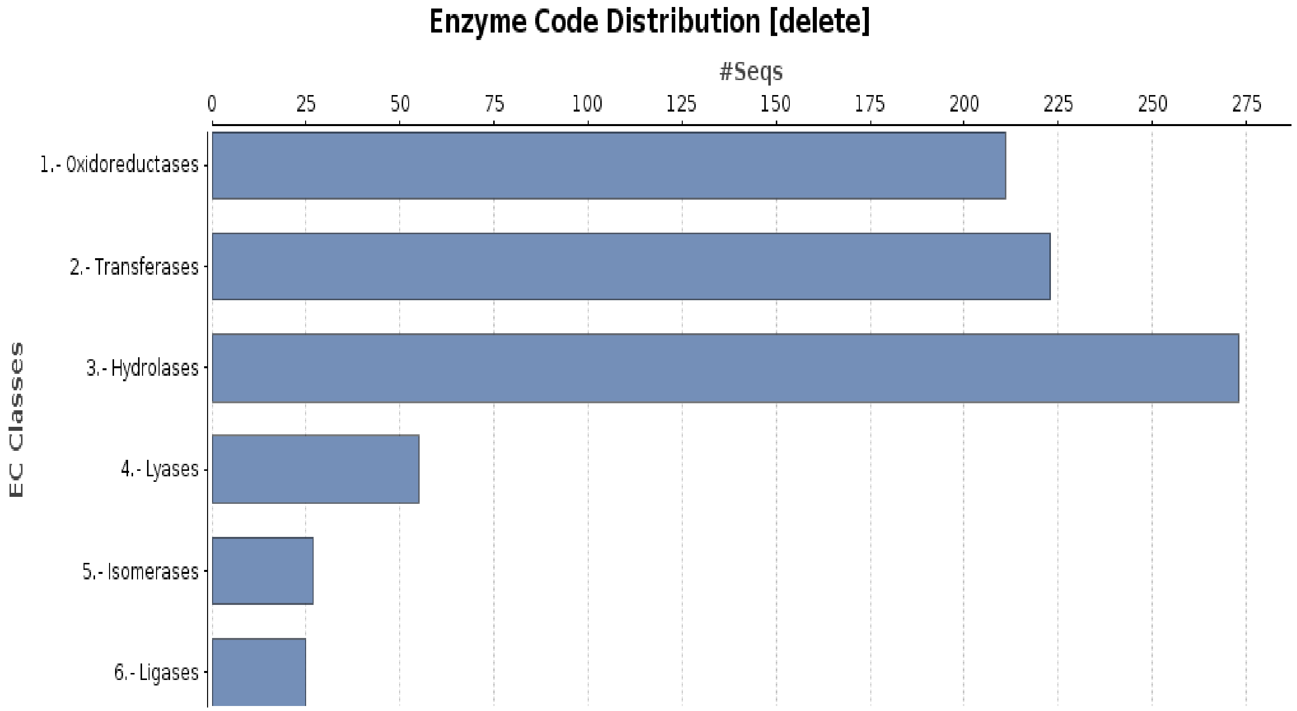

Supplementary Figure 2: Enzyme code distribution for the annotated sequences.

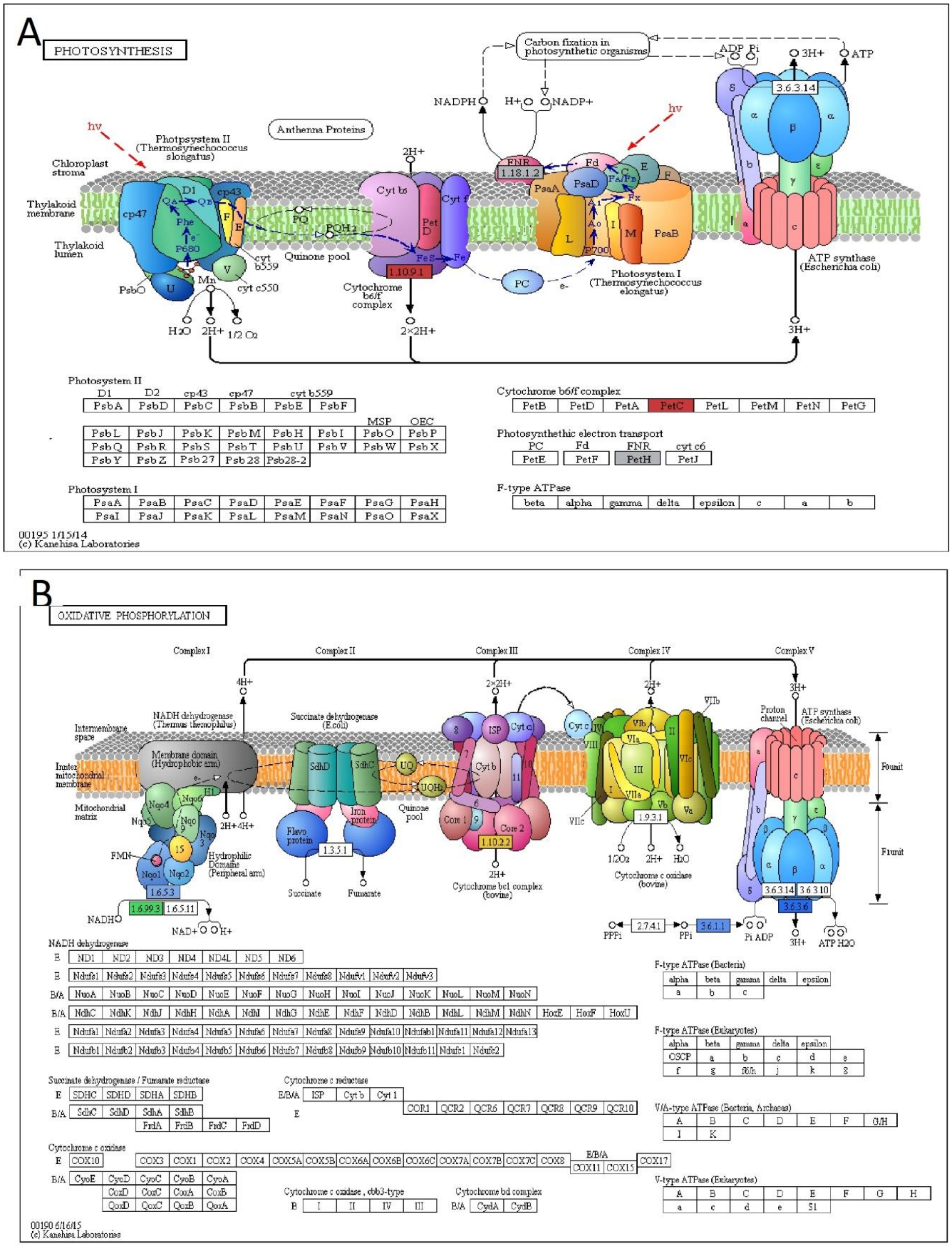

Supplementary Figure 3: CDS co-expressing with lncRNAs and coding for enzymes (indicated by shaded boxes) involved in (A) photosynthesis and (B) oxidative phosphorylation.

The images have been obtained using KEGG[1] map module in BLAST2GO[2] software (https://www.blast2go.com/).

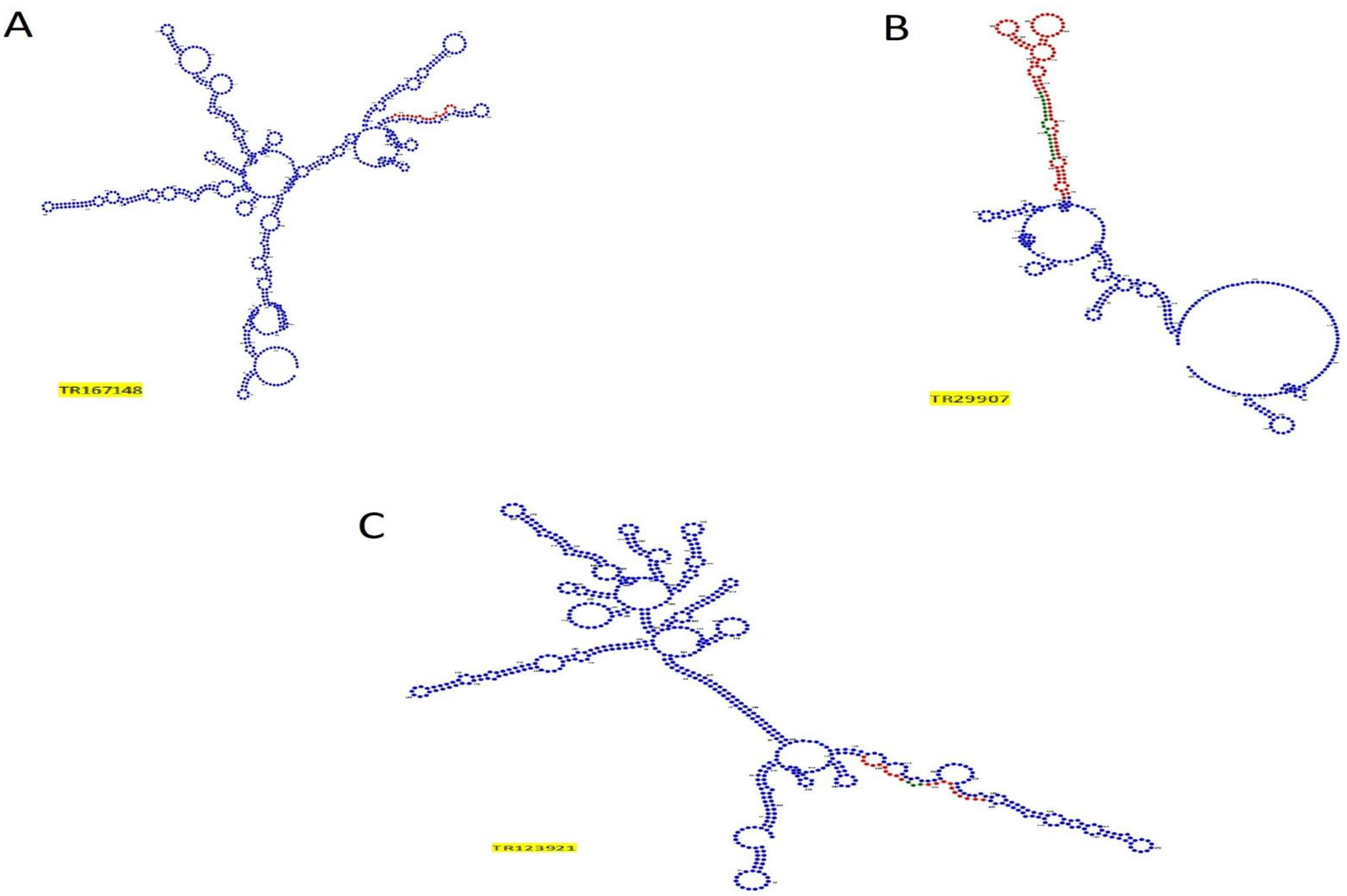

Supplementary Figure 4: The complete secondary structures of lncRNAs (A) TR167148, (B) TR29907, and (C) TR123921.

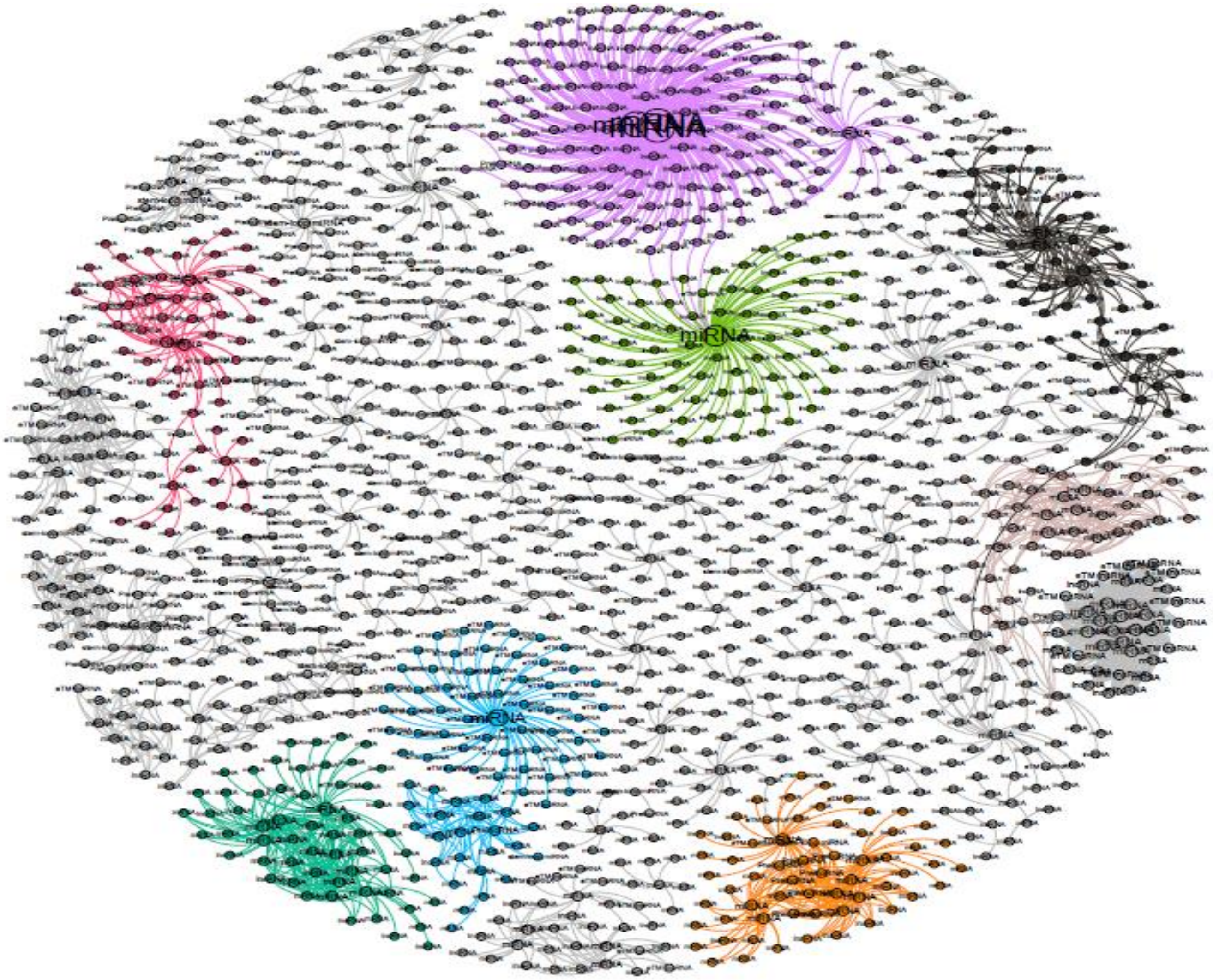

Supplementary Figure 5: The interactome comprising lncRNAs, miRNAs, and mRNAs based on their interactions as putative targets, target mimics, and precursors

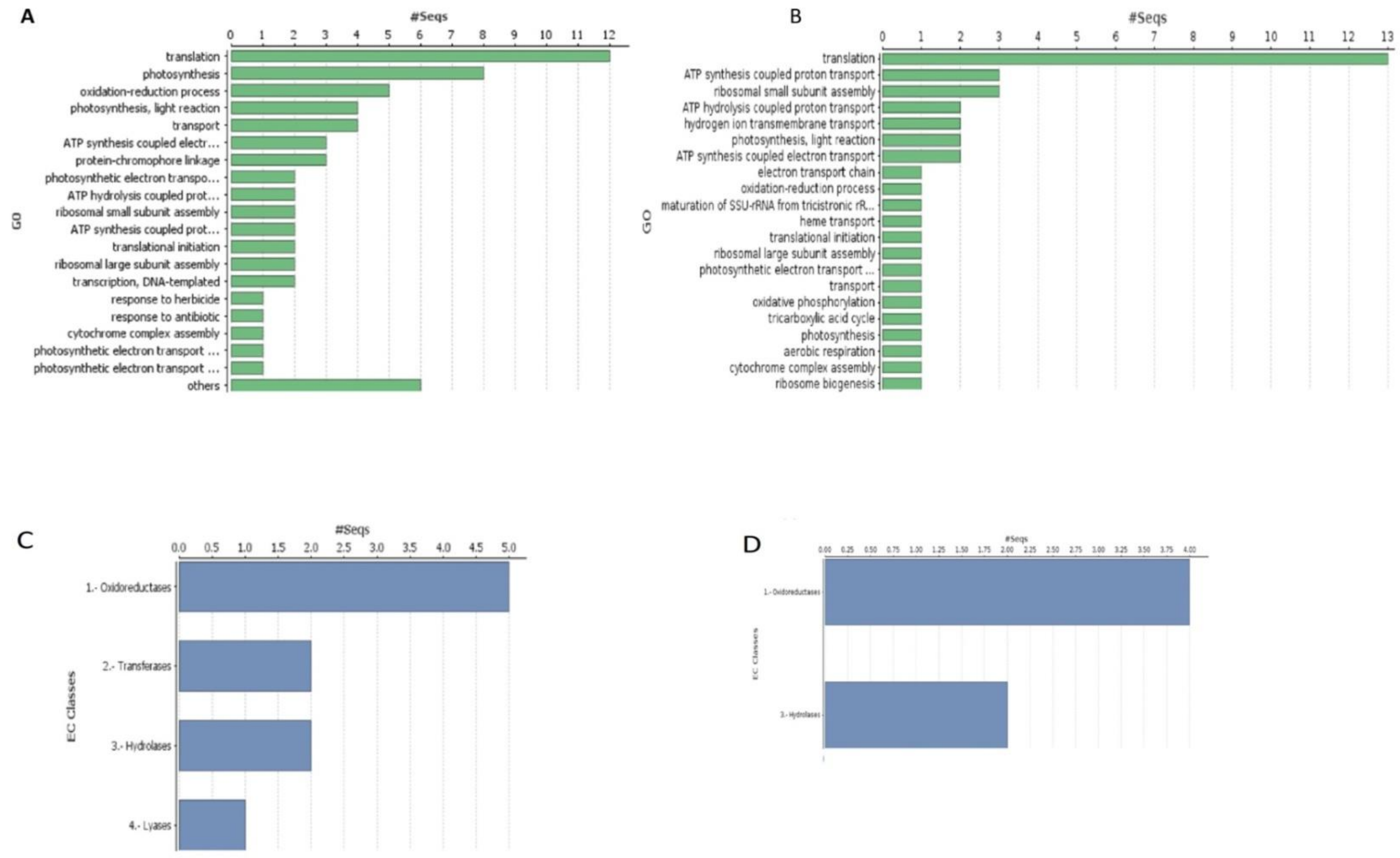

Supplementary Figure 6: Top gene ontology (GO) terms representing the main biological processes involving lncRNAs co-expressing with (A) chloroplast and (B) mitochondrion coding sequences. Enzyme code distribution for the annotated sequences that is (C) chloroplast CDS and (D) mitochondrion CDS co-expressing with lncRNAs

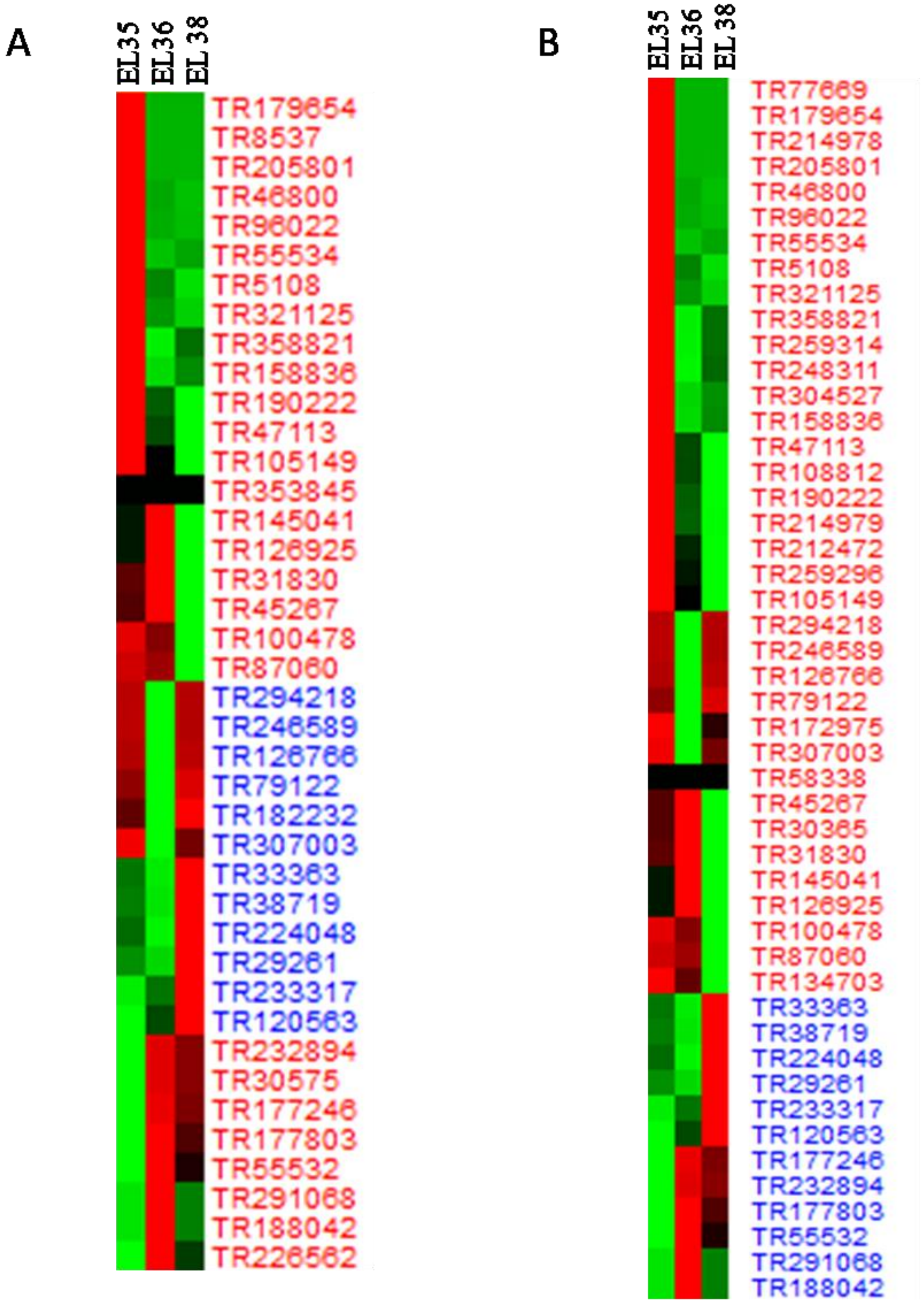

Supplementary Figure 7: Expression patterns of grapevine lncRNAs co-expressing with (A) 9-*cis*-epoxy-carotenoid dioxygenase (NCED) and (B) zeaxanthin epoxidase (ZEP)

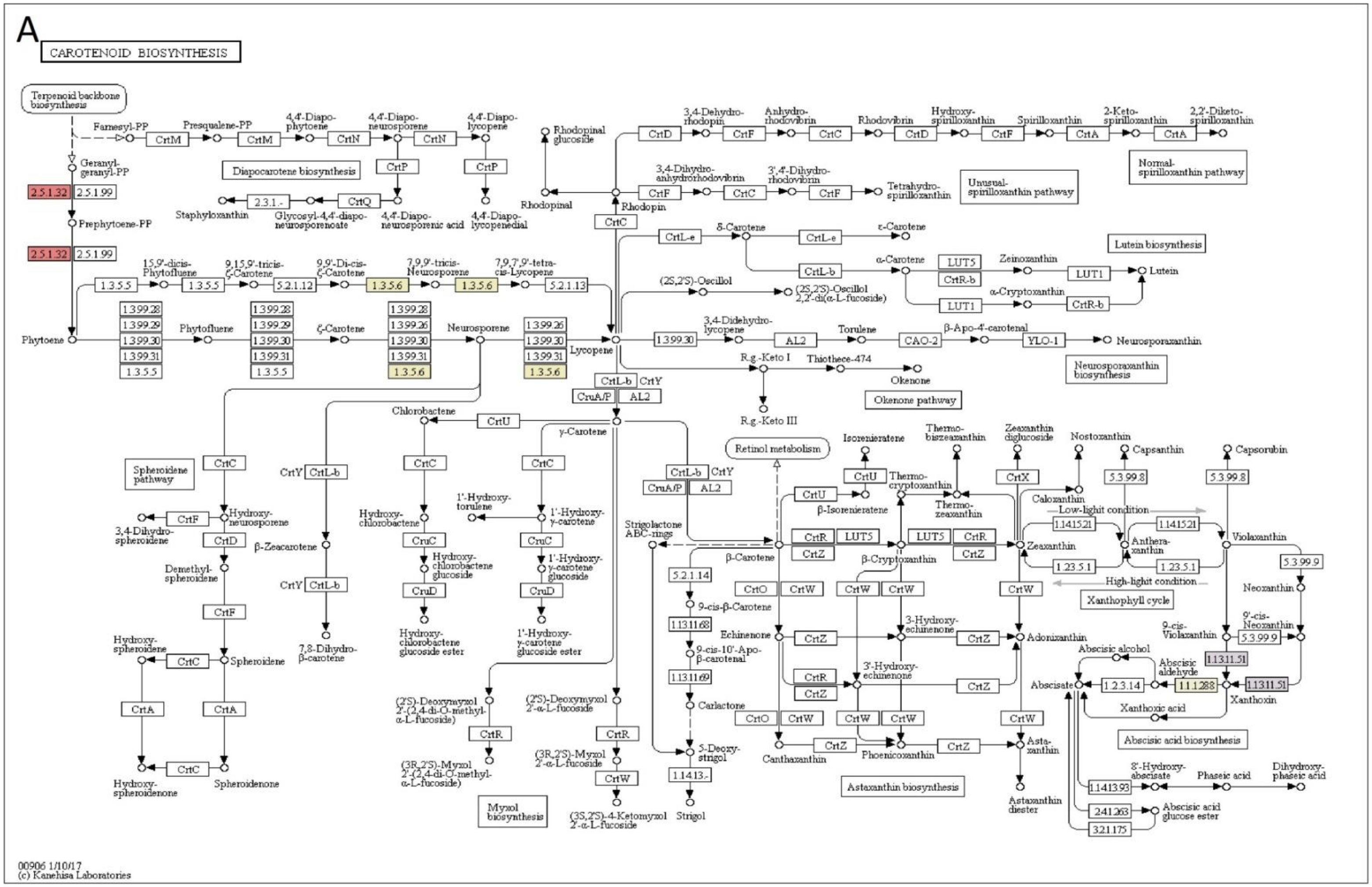

Supplementary Figure 8: CDS co-expressing with lncRNAs and coding for enzymes (indicated by shaded boxes) involved in (A) carotenoid, (B) phenylpropanoid (C) flavonoid, and (D) stilbenoid biosynthesis pathways. The images have been obtained using KEGG[1] map module in BLAST2GO[2] software

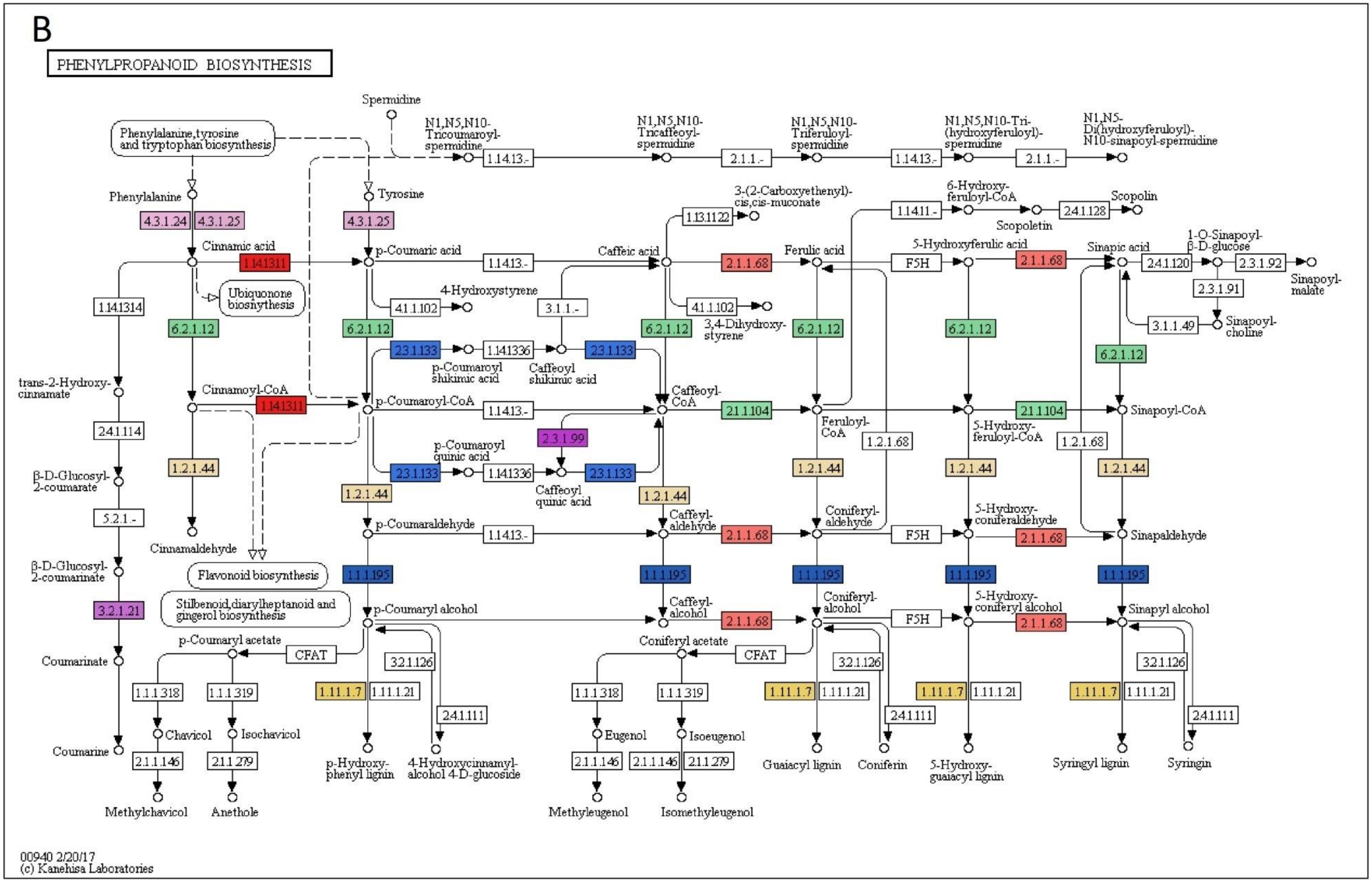

B
PHENYLPROPANOID BIOSYNTHESIS
Phenylalanine,tyrosine and tryptophan biosynthesis
Spermidine
N1,N5,N10-Tricoumaroyl-spermidine
1.14.13.-
N1,N5,N10-Tricaffeoyl-spermidine
2.1.1.-
N1,N5,N10-Triferuloyl-spermidine
1.14.13.-
N1,N5,N10-Tri-(hydroxyferuloyl)-spermidine
2.1.1.-
N1,N5-Di(hydroxyferuloyl)-N10-sinapoyl-spermidine
Phenylalanine
Tyrosine
4.3.1.24
4.3.1.25
4.3.1.25
3-(2-Carboxyethenyl)-cis,cis-muconate
1.13.11.22
6-Hydroxy-feruloyl-CoA
1.14.11.-
Scopoletin
2.4.1.128
Scopolin
Cinnamic acid
1.14.13.11
p-Coumaric acid
1.14.13.-
Caffeic acid
2.1.1.68
Ferulic acid
F5H
5-Hydroxyferulic acid
2.1.1.68
Sinapic acid
2.4.1.120
1-O-Sinapoyl-β-D-glucose
2.3.1.92
Sinapoyl-malate
2.3.1.91
Ubiquonone biosynthesis
4.1.1.102
4-Hydroxystyrene
3.1.1.-
4.1.1.102
3,4-Dihydroxy-styrene
3.1.1.49
Sinapoyl-choline
1.14.13.14
6.2.1.12
6.2.1.12
6.2.1.12
6.2.1.12
6.2.1.12
6.2.1.12
2.3.1.133
p-Coumaroyl shikimic acid
1.14.13.36
Caffeoyl shikimic acid
2.3.1.133
trans-2-Hydroxy-cinnamate
Cinnamoyl-CoA
1.14.13.11
p-Coumaroyl-CoA
1.14.13.-
Caffeoyl-CoA
2.1.1.104
Feruloyl-CoA
5-Hydroxy-feruloyl-CoA
2.1.1.104
Sinapoyl-CoA
2.4.1.114
2.3.1.99
p-Coumaroyl quinic acid
1.2.1.68
1.2.1.68
β-D-Glucosyl-2-coumarate
1.2.1.44
2.3.1.133
1.14.13.36
Caffeoyl quinic acid
2.3.1.133
1.2.1.44
1.2.1.44
1.2.1.44
1.2.1.44
1.2.1.44
5.2.1.-
Cinnamaldehyde
p-Coumaraldehyde
1.14.13.-
Caffeyl-aldehyde
2.1.1.68
Coniferyl-aldehyde
F5H
5-Hydroxy-coniferaldehyde
2.1.1.68
Sinapaldehyde
β-D-Glucosyl-2-coumarinate
Flavonoid biosynthesis
1.1.1.195
1.1.1.195
1.1.1.195
1.1.1.195
1.1.1.195
3.2.1.21
Stilbenoid,diarylheptanoid and gingerol biosynthesis
p-Coumaryl alcohol
Caffeyl-alcohol
2.1.1.68
Coniferyl-alcohol
F5H
5-Hydroxy-coniferyl alcohol
2.1.1.68
Sinapyl alcohol
Coumarinate
p-Coumaryl acetate
CFAT
Coniferyl acetate
CFAT
3.2.1.126
3.2.1.126
3.2.1.126
1.1.1.318
1.1.1.319
1.11.1.7
1.11.1.21
1.1.1.318
1.1.1.319
1.11.1.7
1.11.1.21
1.11.1.7
1.11.1.21
1.11.1.7
1.11.1.21
2.4.1.111
2.4.1.111
2.4.1.111
Chavicol
Isochavicol
Eugenol
Isoeugenol
Coumarine
2.1.1.146
2.1.1.279
p-Hydroxy-phenyl lignin
4-Hydroxycinnamyl-alcohol 4-D-glucoside
2.1.1.146
2.1.1.146
2.1.1.279
Guaiacyl lignin
Coniferin
5-Hydroxy-guaiacyl lignin
Syringyl lignin
Syringin
Methylchavicol
Anethole
Methyleugenol
Isomethyleugenol
00940 2/20/17
(c) Kanehisa Laboratories

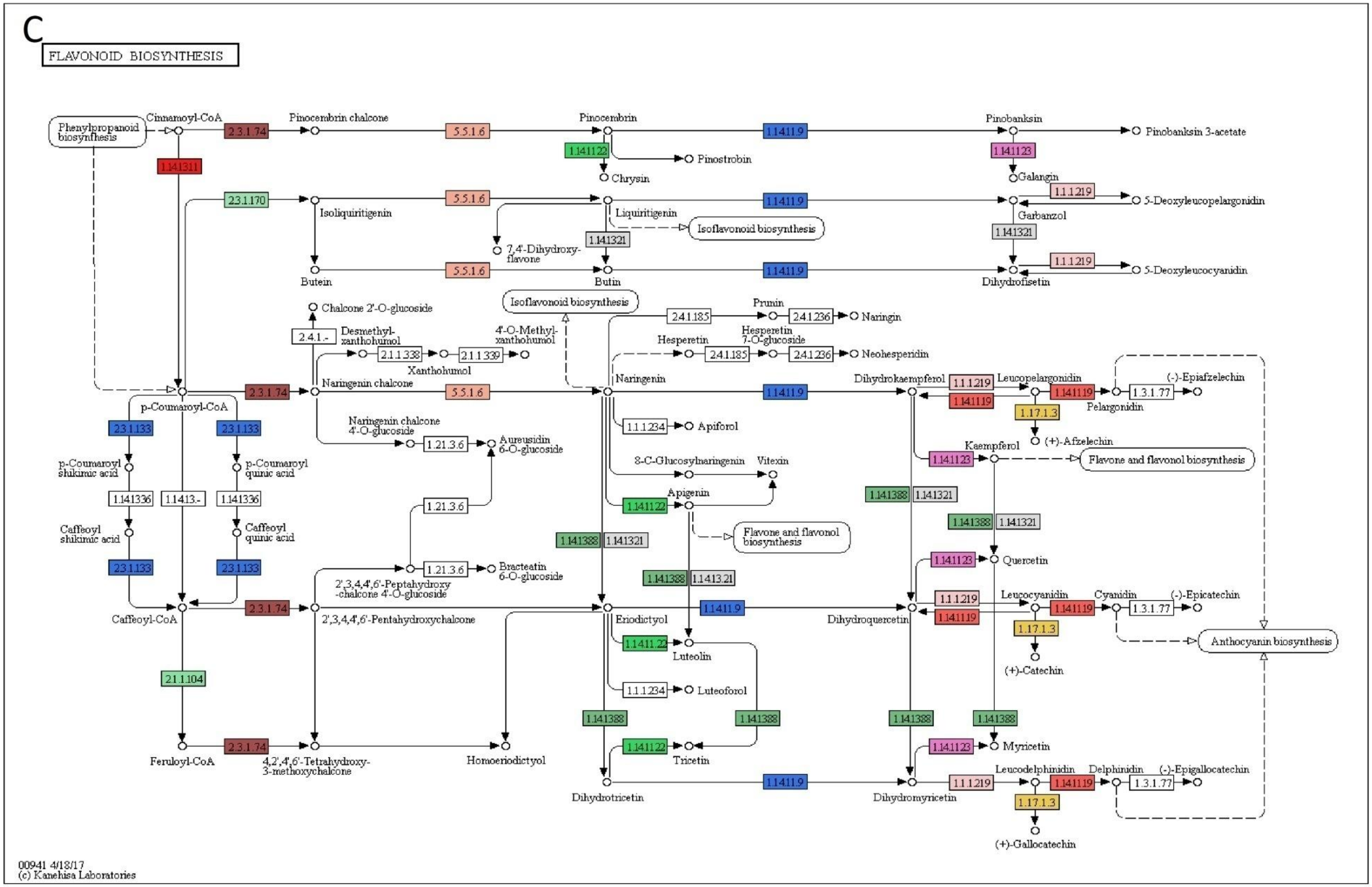
C
FLAVONOID BIOSYNTHESIS
Phenylpropanoid biosynthesis
Cinnamoyl-CoA
Pinocembrin chalcone
Pinocembrin
Pinobanksin
Pinobanksin 3-acetate
Pinostrobin
Chrysin
Galangin
Isoliquiritigenin
Liquiritigenin
Isoflavonoid biosynthesis
7,4'-Dihydroxy-flavone
5-Deoxyleucopelargonidin
Garbanzol
Butein
Butin
Dihydrofisetin
5-Deoxyleucocyanidin
Chalcone 2'-O-glucoside
Desmethyl-xanthohumol
Xanthohumol
4'-O-Methyl-xanthohumol
Prunin
Naringin
Hesperetin
Hesperetin 7-O-glucoside
Neohesperidin
Naringenin chalcone
Naringenin
Dihydrokaempferol
Leucopelargonidin
Pelargonidin
(-)-Epiafzelechin
(+)-Afzelechin
p-Coumaroyl-CoA
p-Coumaroyl shikimic acid
p-Coumaroyl quinic acid
Naringenin chalcone 4'-O-glucoside
Aureusidin 6-O-glucoside
Apiforol
8-C-Glucosylnaringenin
Vitexin
Kaempferol
Flavone and flavonol biosynthesis
Apigenin
Caffeoyl shikimic acid
Caffeoyl quinic acid
Quercetin
2',3,4,4',6'-Peptahydroxy-chalcone 4'-O-glucoside
Bracteatin 6-O-glucoside
Caffeoyl-CoA
2',3,4,4',6'-Pentahydroxychalcone
Eriodictyol
Dihydroquercetin
Leucocyanidin
Cyanidin
(-)-Epicatechin
Anthocyanin biosynthesis
Luteolin
(+)-Catechin
Luteoforol
Feruloyl-CoA
4,2',4',6'-Tetrahydroxy-3-methoxychalcone
Homoeriodictyol
Tricetin
Myricetin
Dihydrotricetin
Dihydromyricetin
Leucodelphinidin
Delphinidin
(-)-Epigallocatechin
(+)-Gallocatechin
2.3.1.74
1.14.13.11
2.3.1.170
5.5.1.6
1.14.11.22
1.14.11.9
1.14.11.23
1.1.1.219
1.14.13.21
2.4.1.-
2.1.1.338
2.1.1.339
2.4.1.185
2.4.1.236
2.3.1.133
1.14.13.36
1.14.13.-
1.21.3.6
1.1.1.234
1.14.11.19
1.17.1.3
1.3.1.77
1.14.13.88
2.1.1.104
00941 4/18/17
(c) Kanehisa Laboratories

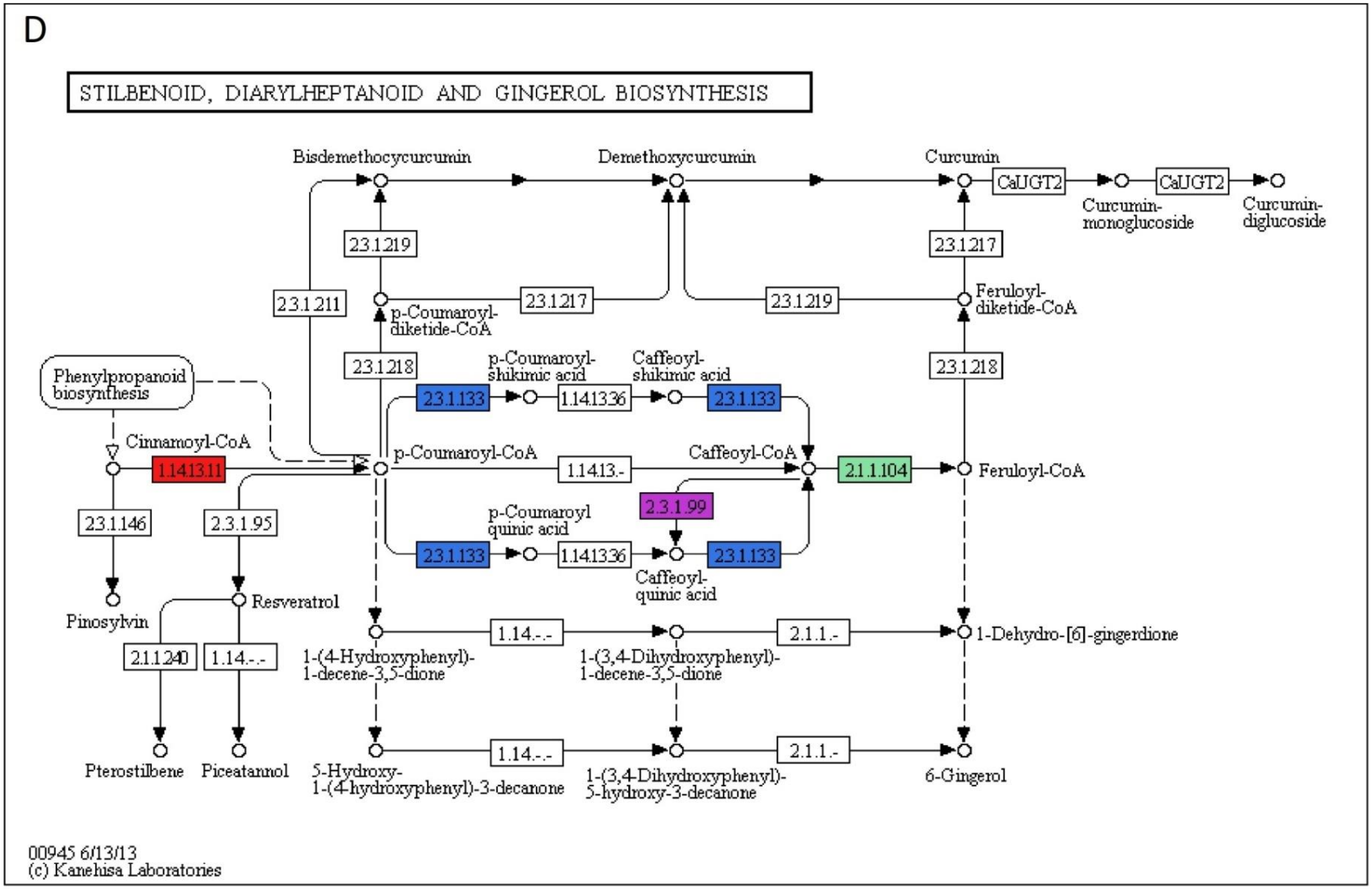
D
STILBENOID, DIARYLHEPTANOID AND GINGEROL BIOSYNTHESIS
Bisdemethocycurcumin
Demethoxycurcumin
Curcumin
CaUGT2
CaUGT2
Curcumin-monoglucoside
Curcumin-diglucoside
2.3.1.219
2.3.1.217
2.3.1.211
2.3.1.217
2.3.1.219
p-Coumaroyl-diketide-CoA
Feruloyl-diketide-CoA
2.3.1.218
2.3.1.218
Phenylpropanoid biosynthesis
p-Coumaroyl-shikimic acid
Caffeoyl-shikimic acid
2.3.1.133
1.14.13.36
2.3.1.133
Cinnamoyl-CoA
p-Coumaroyl-CoA
Caffeoyl-CoA
1.14.13.11
1.14.13.-
2.1.1.104
Feruloyl-CoA
2.3.1.99
p-Coumaroyl quinic acid
2.3.1.146
2.3.1.95
2.3.1.133
1.14.13.36
2.3.1.133
Caffeoyl-quinic acid
Resveratrol
Pinosylvin
1.14.-.-
2.1.1.-
1-Dehydro-[6]-gingerdione
2.1.1.240
1.14.-.-
1-(4-Hydroxyphenyl)-1-decene-3,5-dione
1-(3,4-Dihydroxyphenyl)-1-decene-3,5-dione
1.14.-.-
2.1.1.-
Pterostilbene
Piceatannol
5-Hydroxy-1-(4-hydroxyphenyl)-3-decanone
1-(3,4-Dihydroxyphenyl)-5-hydroxy-3-decanone
6-Gingerol
00945 6/13/13
(c) Kanehisa Laboratories

References for Supplementary Information:

1. Kanehisa, M., Furumichi, M., Tanabe, M., Sato, Y., and Morishima, K.; KEGG: new perspectives on genomes, pathways, diseases and drugs. *Nucleic Acids Research*, 45, D353-D361 (2017)
2. Conesa, A. and Götz, S. Blast2GO: A comprehensive suite for functional analysis in plant genomics. *International Journal of Plant Genomics*, *2008* (2008).
